# A cellular-meso-macro three-scale approach captures remodelling of cancellous bone in health and disease

**DOI:** 10.1007/s10237-025-01948-5

**Published:** 2025-05-03

**Authors:** Areti Papastavrou, Peter Pivonka, Ina Schmidt, Paul Steinmann

**Affiliations:** 1 Faculty of Mechanical Engineering, Technische Hochschule Nürnberg Georg Simon Ohm, Nuremberg, Germany; 2 School of Mechanical, Medical and Process Engineering, Queensland University of Technology, Brisbane, Australia; 3 Institute of Applied Mechanics, Friedrich-Alexander-Universität Erlangen-Nürnberg, Erlangen, Germany; 4 Glasgow Computational Engineering Centre, Glasgow University, Glasgow, UK

**Keywords:** Mechanobiologically-induced remodelling of cancellous bone, Multiscale bone remodelling, Cancellous meso architecture, Macro scale continuum approach, Three-scale approach, Bone cell population model

## Abstract

Remodelling of cancellous bone due to the combined activity of osteoclasts and osteoblasts at the cellular scale has notable repercussions both at the meso (tissue) as well as the macro (organ) scale. At the meso scale, trabeculae adapt their geometry, typically in terms of their cross section, whereas the nominal bone density evolves at the macro scale, all in response to habitual mechanical loading and its perturbations. To capture this intricate scale coupling, we here propose a novel conceptual three-scale approach to the remodelling of cancellous bone. Therein, we combine a detailed bone cell population model at the cellular scale with an idealised trabecular truss network model with adaptive cross sections, that are driven by the cell population model, at the meso scale, which is eventually upscaled to a continuum bone density adaption model at the macro scale. Algorithmically, we solve the meso and macro problems concurrently within a finite element setting and update the cell activity in a staggered fashion. Our benchmark simulations demonstrate the applicability and effectivity of the three-scale approach to analyse bone remodelling in health and disease (here exemplified for the example of osteoporosis) with rich details, e.g. evolving anisotropy, resolved at each scale.

## Introduction

Bone is a complex hierarchically arranged material which is constantly changing due to mechanical demands as well as hormonal changes and/or degenerative diseases as evidenced by in-vivo measurements, see, e.g. Schulte et al. (2013); Christen et al. (2014); Scheuren et al. (2020). On the cellular scale active osteoclasts and active osteoblasts work in a coupled process of bone resorption and bone formation, driving the remodelling process. Consequently, on a so-called meso (tissue) scale, the size and shape of individual trabeculae change, which on the macro (organ) scale can be observed as a change in bone density and evolving effective anisotropy. Physiological exercise which induces macro scale loading then again affects the stress/strain state in the meso structure and thus influences the bone cell activities and the remodelling process at the cellular scale. To properly understand and simulate the bone remodelling process, it is therefore of major importance to account for all of these different scales (Webster and Müller 2011; Garcia-Aznar et al. 2021; Hellmich et al. 2022).

The large variety of computational approaches to bone remodelling can be broadly divided into (i) mechanics-based bone adaptation models (Huiskes et al. 1987; Harrigan and Hamilton 1993; Jacobs et al. 1995; Weinans et al. 1992; Kuhl and Steinmann 2003a, b; DiCarlo et al. 2006; Kaczmarczyk and Pearce 2011; Martin et al. 2020; Papastavrou et al. 2020, 2020) and (ii) mechanobiological bone adaptation models (Lemaire et al. 2004; Buenzli et al. 2012; Pivonka et al. 2008, 2012, 2013; Scheiner et al. 2013). The former models are conceptually simple and are based on purely mechanical quantities such as principal strain, energy storage density, etc. to phenomenologically simulate the evolution of trabecular structure (Meslier and Shefelbine 2023). Within the realm of the former class of phenomenological models to bone, remodelling various sophistications have been proposed recently. As examples we mention, among many others, for instance (George et al. 2020) focusing on the effect of various intensities of the mechanical stimulus on bone cell activity, whereby for medium intensity loads the bone density increases, whereas for high intensity load bone degradation occurs. Giorgio et al. (2016) sets up a visco-poroelastic approach, wherein the stimulus for bone remodelling, motivated by mechano-sensing osteocytes, is of nonlocal, integral type postulated as a combination of the energy storage density and the viscous dissipation. Alternatively, Giorgio et al. (2019, 2023) consider the stimulus for bone remodelling as diffusing in the bone tissue, i.e. satisfying a parabolic evolution equation with first-order derivative in time and second-order derivative in space as well as source and sink terms driven by the energy storage density and the stimulus, respectively. Thereby, Giorgio et al. (2023) in particular propose an orthotropic strain gradient approach to bone remodelling. The latter models are significantly more complex and aim to couple biological processes regulating bone remodelling and mechanical regulatory pathways that give rise to mechanobiological feedback. This increase in model complexity allows to address basic science and clinical bone research questions including effective osteoporosis treatment strategies or efficacy of different drug treatments. However, one major drawback of these models is that due to their complexity and computational costs these have typically been applied only to representative volume elements which essentially results into temporal-only models. Some exceptions are the spatio-temporal models of Hambli et al. (2011) and Lerebours et al. (2016).

The meso structural morphology of trabecular bone gives rise to anisotropic material behaviour observed for different mechanical testing configurations (Woo et al. 2007; Chen et al. 2017; Du et al. 2021). An important feature of trabecular bone is that it can change its effective anisotropy due to remodelling and adaption processes. Most models of trabecular bone remodelling track porosity (bone volume fraction) changes only, and consequently, are not able to predict changes in anisotropy. Only few models have been formulated that can take into account both, i.e. porosity change and, nevertheless merely phenomenologically, changes in anisotropy, e.g. Jacobs et al. (1997); Goda and Ganghoffer (2018); Martin et al. (2020). Taken together, to date no mechanobiologically driven model exists that considers anisotropy induced by changes in the explicitly resolved trabecular meso structure.

In the current contribution, we propose a three-scale approach enabling simulation of bone diseases and drug treatments of trabecular bone accounting for both density changes and effective anisotropy. This model is an extension of previous work of the authors on a two-scale (marco-meso) phenomenological bone remodelling approach (Steinmann et al. 2024) that is here enhanced by a detailed bone cell population model (BCPM) (Scheiner et al. 2013). The two major novelties of our approach are: (i) the BCPM is applied for the adaption of individual trabeculae of the here-called zoom-in volume element (ZVE) at the meso scale and (ii) the ZVE contains a truss network structure which, as part of the bone remodelling process, can change its effective anisotropy. We demonstrate the predictive capabilities of this novel mechanobiological model of trabecular bone remodelling by considering the classical 2D femur adaptation problem introduced by Carter and Beaupré (2001) and simulating osteoporosis in the femur with different parameter configuration of the mechanostat model. As a potential and particular benefit of our approach, the BCPM employed provides various ports to externally inject growth factors, proteins and hormones, for example, to mimick drug dosages and/or diseases.

## Three-scale approach

Existing multi-scale approaches predicting the mechanical response and properties of bone range from the cellular to the organ scale (Hamed et al. 2010; Kwon 2018; Alizadeh et al. 2020; Cen et al. 2021). However, these models exclusively consider the mechanical behaviour and typically do not account explicitly for the intricate trabecular structure of cancellous bone. Exceptions that take the detailed trabecular architecture into account are pixel- and voxel-based models in 2D and 3D as well as $\mu$FEA analysing small cancellous bone specimens regarding their mechanical behaviour, see Jang and Kim (2008); Tsubota et al. (2009); Webster and Müller (2011); Christen et al. (2013); Oliviero et al. (2021). Especially when generated using $\mu$CT or HR-pQCT measurements, the resulting models prove to be particularly true to the real geometry (Woo et al. 2007; Chen et al. 2017; Du et al. 2021). Some $\mu$CT-generated voxel-based finite element models even allow for detailed simulation of the remodelling of small trabecular bone specimens, see e.g, Wang et al. (2014). Nevertheless, the computational cost of fully resolved cancellous bone models is prohibitively expensive for full bone samples. Noteworthy, to increase computational efficiency, Hambli et al. (2011) proposed, for a given trabecular structure characterised by $\mu$CT, a bone remodelling approach that couples finite elements at the macro scale with a neural network trained by voxel-based finite element analyses at the meso scale. Alternatively, some contributions propose using simplified geometries, but either do not consider the remodelling process (Cervantes et al. 2010; Kadir et al. 2010), or only consider a single dedicated trabecular structure (Goda and Ganghoffer 2018). Other studies use simplified trabecular architectures, e.g. through modelling each trabecula as a one-dimensional structural element, however these are not adaptive (Marzban et al. 2015; Phillips et al. 2015). Optimisation-type approaches to incorporate the multi-scale nature of bone determine the trabecular structure by solving a material distribution problem based on density design variables at the macro and meso scale, see Coelho et al. (2009); Fernandes et al. (2012); Wierszycki et al. (2014). Another recent study presents a two-scale model that investigates the nonlinear viscoelastic behaviour of a single trabecula at the meso scale and a bone sample at the macro scale (Jankowski et al. 2023). One of the first approaches to mathematically describe the nonlinear autoregulation between osteoblasts and osteoclasts was presented by Komarova et al. (2003). Nevertheless, most established approaches account only for the biomechanical aspects of bone remodelling, thereby neglecting its mechanobiological foundations.

**Fig. 1:**
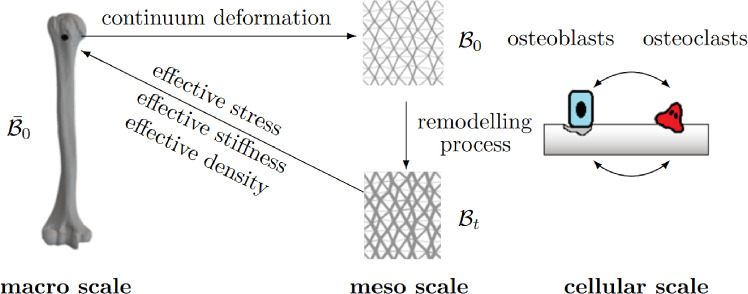
Sketch of three-scale approach: three-scale approach: a bone cell population model for the activity of osteoclasts and osteoblasts at the cellular scale informs the adaptation of the trabecular geometry at the meso scale. Mechanobiological feedback is accounted for via the energy storage density in trabeculae, which regulates activity of osteoblasts and osteoclasts. Effective continuum quantities at the macro scale, i.e. the stress, stiffness and density, follow via upscaling from the meso scale

Recently, we proposed a two-scale (meso-macro scale) approach to computationally capture cancellous bone remodelling, however still based on a phenomenological evolution law for the trabeculae’s cross sections at the meso scale (Steinmann et al. 2024). In the present work, we extend our previous, purely phenomenological two-scale approach to a mechanobiologically inspired three-scale approach (see Fig. 1), where mechano-adaptive mechanisms are considered at the macro and meso scale, respectively, whereas the activity of osteoclast and osteoblast cells is captured at the cellular scale. The kinematics and kinetics at the macro scale are based on a phenomenological continuum bone remodelling approach, see, e.g. Kuhl and Steinmann (2003b). Yet, in our multi-scale approach, the macro scale constitutive behaviour is not postulated phenomenologically but rather follows directly through upscaling from the meso scale Steinmann et al. (2024). At the meso scale, for the sake of demonstration and without loss of generality, the cancellous structure is here idealised as a trabecular truss network. As the key novelty and different to our previous approach, a mechanobiological bone cell population model at the cellular scale ((Buenzli et al. 2012; Pivonka et al. 2008, 2012, 2013; Scheiner et al. 2013)) drives the adaptation of the trabeculae’s cross sections, thereby the bone cell population model is coupled to the concurrently solved mechano-adaptive macro-meso problems in a staggered fashion.

### Macro scale continuum model

At the macro scale, we consider bone as continuous matter (Kuhl and Steinmann 2003b; Papastavrou et al. 2020, 2020; Schmidt et al. 2021, 2022), i.e. in terms of *effective* continuum quantities that do not explicitly resolve any sub (meso, cellular) scale features. We here adopt a geometrically nonlinear continuum formulation for the sake of modelling rigour; nevertheless, for the range of deformations expected for hard bone tissue exposed to habitual mechanical loading, the response will automatically approach the geometrically linear limit. All continuum quantities at the macro scale are indicated by an overbar.

*Kinematics:* The kinematics at the macro scale are characterised by the nonlinear deformation map $\bar{\varvec{y}}$ relating the placement $\bar{\varvec{X}}$ of a continuum point in the material (reference) configuration $\bar{\mathcal {B}}_{0}$ to its position $\bar{\varvec{x}}$ in the spatial (deformed) configuration $\bar{\mathcal {B}}_{t} \subset \mathbb {E}^3$

1 $$\begin{aligned} \bar{\varvec{x}} = \bar{\varvec{y}}(\bar{\varvec{X}},{\bar{t}}): \bar{\mathcal {B}}_0 \times \mathbb {R}_{+} \rightarrow \bar{\mathcal {B}}_{t} \,. \end{aligned}$$

Here ${\bar{t}}$ denotes the macro time scale (here, for the sake of simplicity, but without loss of generality, coinciding with the meso time scale *t*) that is typically much larger than the cellular time scale (as reflected by the different time step sizes in the computational examples, see below). The material gradient of the deformation map is denoted the deformation gradient $\bar{\varvec{F}}$

2 $$\begin{aligned} \bar{\varvec{F}} = \overline{\operatorname {Grad}} \bar{\varvec{y}}(\bar{\varvec{X}},{\bar{t}}): T\bar{\mathcal {B}}_0 \rightarrow T\bar{\mathcal {B}}_{t} \,, \end{aligned}$$

The differential operator $\overline{\operatorname {Grad}}$ expresses the gradient in terms of derivatives with respect to the material coordinates $\bar{\varvec{X}}$. The deformation gradient $\bar{\varvec{F}}$, a two-point tensor, linearly maps from the material tangent space $T\bar{\mathcal {B}}_{0}$ to the spatial tangent space $T\bar{\mathcal {B}}_{t}$.

*Kinetics:* The kinetics at the macro scale is collectively dictated by the balances of mass and linear momentum

3 $$\begin{aligned} \dot{\bar{\rho }}_0 ={\bar{R}}_0\quad \text{ and }\quad \overline{\operatorname {Div}} \bar{\varvec{P}} = \varvec{0} \,, \end{aligned}$$

with the nominal mass density $\bar{\rho }_0$ per unit volume in $\bar{\mathcal {B}}_0$, corresponding mass source ${\bar{R}}_0$, and Piola stress $\bar{\varvec{P}}$ (a two-point tensor mapping from $T^*\bar{\mathcal {B}}_0$ to $T^*\bar{\mathcal {B}}_t$, the material and spatial cotangent spaces), respectively. The differential operator $\overline{\operatorname {Div}}$ expresses the divergence in terms of derivatives with respect to the material coordinates $\bar{\varvec{X}}$.

Body forces and inertia are here neglected due to the different levels of gravitational and habitual mechanical loading as well as due to the different time scales of the macro scale problem and the bone remodelling process at the cellular scale.

*Constitutive Relations:* A traditional one-scale phenomenological bone remodelling approach (Kuhl and Steinmann 2003b; Papastavrou et al. 2020, 2020; Schmidt et al. 2021, 2022) requires a constitutive model that expresses the mass source and the Piola stress in terms of the nominal mass density and the deformation gradient, i.e.

4 $$\begin{aligned} {\bar{R}}_0={\bar{R}}_0(\bar{\rho }_0, \bar{\varvec{F}})\quad \text{ and }\quad \bar{\varvec{P}}=\bar{\varvec{P}}(\bar{\rho }_0, \bar{\varvec{F}})\,. \end{aligned}$$

In our multi-scale approach, the density evolution in Eq. 3.1 (and thus the constitutive model in Eq. 4.1) as well as the constitutive model in Eq. 4.2 are however entirely by-passed by resorting to *upscaling* from the meso scale (see below)

5 $$\begin{aligned} \bar{\rho }_0=\langle \rho _0\rangle \ \text{ and } \ \bar{\varvec{P}}=\langle \varvec{P}\rangle \ \text{ with } \ \bar{\varvec{A}}=\langle \varvec{A}\rangle \,. \end{aligned}$$

Here the macro scale quantities $\bar{\rho }_0$ and $\bar{\varvec{P}}$ together with its consistent linearisation $\bar{\varvec{A}}$ are equated with corresponding averaged values $\langle \rho _0\rangle$, $\langle \varvec{P}\rangle$ and $\langle \varvec{A}\rangle$ at the meso scale. The determination of $\langle \rho _0\rangle$, $\langle \varvec{P}\rangle$ and $\langle \varvec{A}\rangle$ from a meso scale truss network model representing the trabecular architecture is outlined next.

**Remark:** Note that, different to alternative approaches advocated, e.g. in Giorgio et al. (2016, 2019, 2023), our three-scale approach incorporates only the displacement map as primary kinematic variable together with, as a derived quantity, its derivative, the deformation gradient. All other quantities at the macro scale, i.e. the nominal mass density, the Piola stress and its consistent linearisation follow via upscaling from the meso scale. In our approach the various bone cell densities live only on the cellular scale and do not constitute macro scale variables. The stimulus driving the evolution of the various bone cells at the cellular scale is the energy storage as computed from the meso scale, see Sect. 2.2. Taken together, there are no macro scale fields for the bone cell densities and no macro scale evolution equation for the nominal bone density or the bone stiffness tensor. In our view, this represents the beauty of the advocated three-scale approach, which by-passes phenomenology at the macro scale as much as possible.

### Meso scale network model

Each continuum point at the macro scale is assigned a *zoom-in volume element* (ZVE) detailing the trabecular architecture at the meso scale. Accepting a severe geometric simplification, we here consider the trabecular meso structure as an idealised trabecular *truss network*. The restriction to a geometrically linear setting is justified by the small level of external loading (as normalised by the bone stiffness) and in addition entirely avoids rather technical and in the current context unnecessary difficulties when formulating geometrically nonlinear truss networks.

**Remark:** We use the terminology ZVE rather than the common terminology ’representative volume element (RVE)’ to account for the lacking scale separation between the solution domain of a ZVE at the meso scale and a continuum point at the macro scale. Indeed, we accept the lack of scale separation to avoid the need to resolve the entire cancellous part of a bone in all detail at the macro scale, which unfortunately is prohibitive effort-wise and moreover also deemed unnecessary.$\square$

**Remark:** Geometrically, cancellous bone is characterised by an intricate meso architecture consisting of one-, and two- (as well as three-) dimensional subdomains, which, when captured exactly, today still result, however, in a prohibitive computational burden. Resorting to a multi-scale approach allows us generically incorporating the architecture of the trabecular structure at the meso scale and thus requires formulating corresponding evolution equations for its remodelling. Here, for the sake of simplicity and to highlight the methodology, we opt to consider an idealised truss network to characterise the trabecular architecture at the meso scale. In this case, an evolution equation for the truss cross sections, i.e. *geometrical quantities*, is a natural option with the cross-sectional evolution driven by the bone cell population model operating at the underlying cellular scale, see below. We like to stress though that the methodology is not restricted to more involved trabecular architectures at the meso scale such as, e.g. composed by beam-, membrane-, plate-, or shell-like subdomains. Likewise, within individual trabeculae remodelling need not render a uniform change in their geometry. In this regard, the advocated truss network approach is indeed a compromise between accuracy and computational efficiency and is thus considered only a first step towards more realistically capturing the "true" cancellous bone and its adaption to mechanical stimuli. Obviously, any of these extensions come at the expense of way higher computational costs. $\square$

*Kinematics:* In a nutshell, a truss network consists of a set of node points $\mathcal{N}$ (globally) numbered by $a=1,\ldots n_{np}$ connected by elements numbered by $e=1,\ldots n_{el}$. The coordinates and displacements of the node points are $\varvec{X}_a$ and $\varvec{u}_a$, respectively, for all $a=1,\ldots n_{np}$. Further details are outlined in the appendix.

*Statics:* For a linear elastic truss network the total energy storage in one element reads $W^e=E^\textrm{s}A^eL^e[\epsilon ^e]^2/2$. Here, $E^\textrm{s}$ denotes the elastic modulus of the solid material (assumed constant and given for the trabeculae) and $A^e, L^e, \epsilon ^e$ denote the element cross-sectional area (the element cross-sectional area $A^e$ is kept constant within one macro/meso time step $\Delta {\bar{t}}$, but updated from one macro/meso time step to the next as informed by the bone cell population model at the cellular scale, see below), the element length and element strain, respectively. Due to the strain $\epsilon ^e$ being constant in an element, there is no volume integral appearing over the energy storage density $w^e=E^\textrm{s}[\epsilon ^e]^2/2$ per volume, but rather only multiplication by the total volume $V^e=A^eL^e$ of an element. Further details are outlined in the appendix.

Next, for the down- and upscaling from the macro scale, we adopt an approach inspired by computational homogenisation, see, e.g. Saeb et al. (2016) and the extensive reference list therein.

*Downscaling:* To relate the macro to the cellular scale, we impose affine displacement boundary conditions to the ZVE. It is noted that other boundary conditions for the ZVE are indeed possible, among them the limiting cases of Voigt and Reuss bounds, traction boundary conditions, as well as the perhaps most accurate, periodic boundary conditions. For a more in-depth discussion, we refer to Saeb et al. (2016) and references therein. Displacement boundary conditions are here merely chosen for the sake of simplicity and demonstration, however without loss of generality. Both traction and periodic boundary conditions require more technicalities when it comes to their implementation, see, e.g. Saeb et al. (2016) among many other accounts on the matter, but do not pose conceptual difficulties per se. It is acknowledged that the selection of either displacement, periodic, or traction boundary conditions results in an effective upscaled response that is either too stiff, believed to be about right, or too soft.

For affine displacement boundary conditions, the displacements $\varvec{u}_a$ of the truss network node points on the ZVE boundary (constituting the set of boundary node points $\mathcal{N}^\textrm{b}$) are prescribed in terms of the deformation gradient $\bar{\varvec{F}}$ from the macro scale

6 $$\begin{aligned} \varvec{u}_a = \bar{\varvec{F}} \cdot \varvec{X}_a -\varvec{X}_a \quad \forall \; a\in \mathcal{N}^\textrm{b}\,. \end{aligned}$$

With these boundary conditions given, the equilibrium problem is solved for the displacements $\varvec{u}_a$ of the truss network node points within the ZVE, thus completing the downscaling.

*UpScaling:* To relate the meso to the macro scale, first averaged values for the nominal density $\langle \rho _0\rangle$ and the Piola stress $\langle \varvec{P}\rangle$ are computed for the ZVE

7 $$\begin{aligned} \begin{aligned}&\langle \rho _0\rangle = \frac{V_\textrm{T}}{V_\textrm{ZVE}} \, \rho _{0}^\textrm{s}\\&\langle \varvec{P}\rangle = \frac{1}{V_\textrm{ZVE}} \sum _{a\in \mathcal{N}^\textrm{b}} \varvec{f}_a\otimes \varvec{X}_a\,. \end{aligned} \end{aligned}$$

Here $V_\textrm{ZVE}$, $V_\textrm{T}=\sum _{e=1}^{n_{el}}V^e$, $\rho _{0}^\textrm{s}$ and $\varvec{f}_a$ denote the volume occupied by the ZVE, the volume occupied by the trabeculae within the ZVE, the density of the solid material (assumed constant and given for the trabeculae) and the reaction forces (resulting from the assembly of internal forces) at the boundary node points $a\in \mathcal{N}^\textrm{b}$.

Upon linearising $\langle \varvec{P}\rangle$ with respect to $\bar{\varvec{F}}$ so that $\delta \langle \varvec{P}\rangle =\langle \varvec{A}\rangle :\delta \bar{\varvec{F}}$, we eventually obtain the fourth-order stiffness tensor $\langle \varvec{A}\rangle$. A detailed description of $\langle \varvec{A}\rangle$ can be found in the appendix.

Finally, the nominal density $\langle \rho _0\rangle$, the Piola stress $\langle \varvec{P}\rangle$ and its linearisation $\langle \varvec{A}\rangle$ as averaged over the ZVE are equated with their counterparts $\bar{\rho }_0$, $\bar{\varvec{P}}$ and $\bar{\varvec{A}}$ at the macro scale, thus completing the upscaling.

### Cellular scale BCPM

The bone remodelling process is here described following the BCPM (bone cell population modelling) approach proposed by Lemaire et al. (2004) and further refined by Pivonka et al. (2008, 2010); Scheiner et al. (2013). Thereby different types of bone cells involved in bone remodelling (active osteoclasts, $\text{rm}{OC_{a}}$, active osteoblasts, $\mathrm {OB_a}$, their precursors, $\mathrm {OC_p}$ and $\mathrm {OB_p}$, respectively, and uncommitted osteoblasts, $\mathrm {OB_u}$), are linked via various regulatory factors. In the current BCPM, we consider mechanobiological regulations via two mechanisms: (i) anabolic actions via regulation of the proliferation of osteoblast precursor cells $\mathrm {OB_p}$ and (ii) catabolic actions via the RANK-RANKL-OPG signalling pathway.

*Bone Cell Population Model:* We formulate the BCPM as a nonlinear evolution system in coupled ODE format. Therein cells are generated due to *differentiation* with $\mathcal {D}_\mathrm {OB_p}$, $\mathcal {D}_\mathrm {OC_p}$, and $\mathcal {D}_\mathrm {OB_u}$ from precursor and uncommitted cell population pools ($\mathrm {OB_p}$, $\mathrm {OC_p}$, and $\mathrm {OB_u}$) together with mechanically regulated *proliferation* of $\mathrm {OB_p}$ with $\mathcal {P}_\mathrm {OB_p}$ (all with positive sign). Moreover, cells can leave a respective cell pool either undergoing *apoptosis* with $\mathcal {A}_\mathrm {OC_a}$ and $\mathcal {A}_\mathrm {OB_a}$ for $\mathrm {OC_a}$ and $\mathrm {OB_a}$, respectively, or via differentiation of $\mathrm {OB_p}$ with $\mathcal {D}_\mathrm {OB_p}$ (all with negative sign) (Pivonka et al. 2008; Scheiner et al. 2013).

Using matrix–vector notation^1^ we collect active osteoclasts $\mathrm {OC_a}$, precursor osteoblasts $\mathrm {OB_p}$, and active osteoblasts $\mathrm {OB_a}$ into the (column) vector $\textbf{x}=[\mathrm {OC_a}, \mathrm {OB_p}, \mathrm {OB_a}]^\textrm{T}$ of independent (solution) variables as well as precursor osteoclasts $\mathrm {OC_p}$ and uncommitted osteoblasts $\mathrm {OB_u}$ into the (column) vector $\textbf{c}=[\mathrm {OC_p}, \mathrm {OB_u}]^\textrm{T}$ of prescribed (constant) variables. Then, the nonlinear evolution equations of the BCPM read in abbreviated matrix–vector format as

8 $$\begin{aligned} \dot{\textbf{x}}=\textbf{S}(\textbf{x})\textbf{x}+\textbf{f}(\textbf{x}; \textbf{c}). \end{aligned}$$

For the current BCPM, the solution-dependent *system matrix* expands as $\textbf{S}(\textbf{x})=$

$$\begin{aligned}\left[ \begin{matrix} -\mathcal {A}_\mathrm {OC_a}\left( \mathrm {OC_a}\right) & 0& 0\\ 0 & \mathcal {P}_\mathrm {OB_p}\left( w^e\right) -\mathcal {D}_\mathrm {OB_p}\left( \mathrm {OC_a}\right) & 0\\ 0& \hspace{1.78cm}+\mathcal {D}_\mathrm {OB_p}\left( \mathrm {OC_a}\right) & -\mathcal {A}_\mathrm {OB_a} \end{matrix}\right] \end{aligned}$$

with the vector of unknown cell concentrations $\textbf{x}=[\mathrm {OC_a}, \mathrm {OB_p}, \mathrm {OB_a}]^T$ and the energy storage density $w^e$ as a parameter. The corresponding solution-dependent *forcing vector* is linearly parameterised in the prescribed $\textbf{c}$ as $\textbf{f}(\textbf{x}; \textbf{c})=$

$$\begin{aligned} \left[ \begin{array}{cc} \mathcal {D}_\mathrm {OC_p}\left( \mathrm {OB_p}, \mathrm {OB_a}, w^e\right) & 0\\ 0& \mathcal {D}_\mathrm {OB_u}\left( \mathrm {OC_a}\right) \\ 0& 0 \end{array}\right] \left[ \begin{array}{ll} \mathrm {OC_p}\\ \mathrm {OB_u} \end{array}\right] . \end{aligned}$$

Note that the cell concentration of osteoclast precursor cells $\mathrm {OC_p}$ and uncommitted osteoblasts $\mathrm {OB_u}$ collected in $\textbf{c}=[\mathrm {OC_p}, \mathrm {OB_u}]^T$ is here assumed constant (Scheiner et al. 2013).

Mechanobiological feedback is included into the BCPM model considering that mechanical loading leads to changes in the energy storage density $w^e$ experienced in individual trabeculae. Changes in the habitual loading (equilibrium) state characterised by the threshold $w^\textrm{a}$ (the *attractor*) are sensed by the osteocytes embedded in the bone matrix and give rise to anabolic (for the case of overloading) or catabolic (for the case of underloading) regulatory signals. Below we describe the modelling of anabolic and catabolic regulatory actions via $\mathcal {P}_\mathrm {OB_p}$ and $\mathcal {D}_\mathrm {OC_p}$, respectively, the remaining (repressor/activator) functionals $\mathcal {D}_\mathrm {OB_p}$, $\mathcal {D}_\mathrm {OB_u}$, $\mathcal {A}_\mathrm {OC_a}$, and $\mathcal {A}_\mathrm {OB_a}$ are detailed in the appendix.

- *Anabolic feedback*: In the case of *overloading* the proliferation of osteoblast precursor cells $\mathrm {OB_p}$ is regulated via $\mathcal {P}_\mathrm {OB_p}=P_\mathrm {OB_p}\bar{\mathcal {P}}_\mathrm {OB_p}$ with $P_\mathrm {OB_p}$ the maximum proliferation rate, a BCPM parameter, and $\bar{\mathcal {P}}_\mathrm {OB_p}=\bar{\mathcal {P}}_\mathrm {OB_p}(w^e)$ the mechanobiological activator function depending on the energy storage density $w^e$. We follow the approach of Scheiner et al. (2013) and use a piecewise constant-linear-constant function $\frac{1}{2}\le \bar{\mathcal {P}}_\mathrm {OB_p}=\frac{1}{2}\big [1+\lambda [\bar{w}^e-1]\big ]\le 1$ with the *anabolic strength parameter* $\lambda$, another BCPM parameter, and the *relative* stored energy density $1\le {\bar{w}}^e=w^e/w^\textrm{a}\le 1+\lambda ^{-1}=:\bar{w}^e_\textrm{max}$. Thus, the mechanobiological activator function $\bar{\mathcal {P}}_\mathrm {OB_p}$ takes a minimum value of $\frac{1}{2}$ for habitual loading with ${\bar{w}}^e=1$ and a maximum value of 1 in case of overloading with ${\bar{w}}^e\ge {\bar{w}}^e_{\max }$. The value of $\lambda$ determines how strong the anabolic pathway regulates bone cell activity. Indeed, $\lambda ^{-1}={\bar{w}}^e_\textrm{max}-1$ expresses the increase of the relative energy storage density ${\bar{w}}^e$ from its threshold value ${\bar{w}}^e=1$ to its maximum value ${\bar{w}}^e_\textrm{max}$, i.e. how fast $\bar{\mathcal {P}}_\mathrm {OB_p}(w^e)$ saturates to its maximum value of 1. For example $\lambda =1,2,4,\ldots$ renders $\bar{w}^e_\textrm{max}-1=1, \frac{1}{2}, \frac{1}{4}, \ldots$, i.e. larger $\lambda$ result in a smaller difference between $w^\textrm{a}$ and $w^e_\textrm{max}$. Conversely, for very small values of $\lambda$, i.e. for $\lambda \rightarrow 0$ and thus ${\bar{w}}^e_{\max }\rightarrow \infty$, the anabolic mechanical feedback tends to zero.
- *Catabolic feedback*: In the case of *underloading* the differentiation of osteoclast precursor cells $\mathrm {OC_p}$ into active osteoclasts $\mathrm {OC_a}$ is *positively* regulated via $\mathcal {D}_\mathrm {OC_p}=D_\mathrm {OC_p}\bar{\mathcal {D}}_\mathrm {OC_p}$ with $D_\mathrm {OC_p}$ the maximum differentiation rate, a BCPM parameter, and $\bar{\mathcal {D}}_\mathrm {OC_p}$ the *activator* functional depending on the RANKL^2^*activator* function and three further BCPM parameters. The RANKL activator function depends in turn on the precursor and active osteoblasts $\mathrm {OB_p}$, $\mathrm {OB_a}$ (together with implicit dependence on the *osteoprotegerin* $\textrm{OPG}$, the *parathyroid hormone* $\textrm{PTH}$, and the *receptor* $\textrm{RANK}$ alongside numerous further BCPM parameters) and, importantly, on the energy storage density $w^e$, i.e. essentially $\textrm{RANKL}=\textrm{RANKL}\left( \mathrm {OB_p}, \mathrm {OB_a}, w^e\right)$. More specifically, the RANKL activator function is proportional to the RANKL *dosage* with mechanobiologically driven contribution, here modelled by the piecewise linear-constant function $\kappa [1-\bar{w}^e]$, i.e. $\textrm{RANKL}\propto \kappa [1-{\bar{w}}^e]$ with $\kappa$ the *inhibition parameter*, a further BCPM parameter, and $1\ge 1-{\bar{w}}^e\ge 0$. For habitual loading with ${\bar{w}}^e=1$ (and also for overloading with ${\bar{w}}^e>1$) the RANKL dosage vanishes, while for underloading scenarios with $0\le {\bar{w}}^e<1$ the RANKL dosage is positive. Therein the value of $\kappa$ determines how strong the catabolic pathway regulates bone cell activity. In addition to the mechanobiologically driven RANKL dosage the BCPM considers furthermore the external RANKL injection $\textrm{RANKL}_d$ that is independent of the mechanical under- and overloading and thus always positive, i.e. $\textrm{RANKL}_d\ge 0$. In health $\textrm{RANKL}_d=0$ whereby in osteoporosis is driven by $\textrm{RANKL}_d> 0$.

**Fig. 2:**
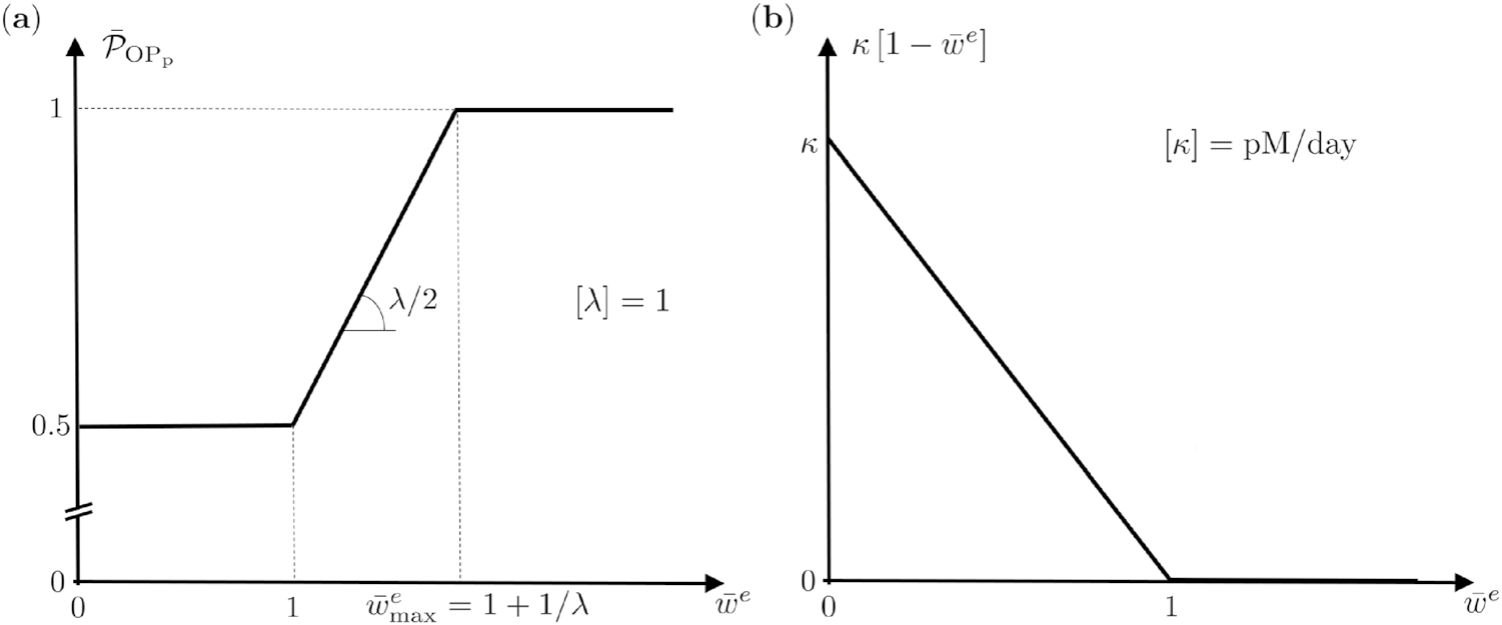
Anabolic and catabolic feedback functions: (**a**) mechanobiological activator function $\bar{\mathcal {P}}_\mathrm {OB_p}$ and (**b**) mechanobiologically driven RANKL dosage function $\kappa [1-\bar{w}^e]$. The unit pM abbreviates picomolar, i.e. 1 pM = $10^{-12}$ mol/L = $10^{-9}$ mol/$\hbox {m}^3$

The functions discussed in the above are displayed in Fig. 2. For a detailed description of the biochemical and mechanobiological signalling pathways together with an outline of the BCPM parameters employed, we refer the reader to Scheiner et al. (2013). Further background information on the BCPM is given in the appendix that also lists some pertinent parameters used in our simulations.

**Remark:** The BCPM provides ports to externally inject for example OPG, PTH, or RANK so as to mimic drug dosages and/or diseases. $\square$

**Remark:** Defining $\textbf{e}=[1, 1, 1]^\textrm{T}$, the temporal evolution of the total bone cell population, i.e. $\textrm{O}=\mathrm {OC_a}+ \mathrm {OB_p}+\mathrm {OB_a}=\textbf{e}^\textrm{T}\textbf{x}$ here reads as

$$\begin{aligned} \begin{aligned} \dot{\textrm{O}}=&-\mathcal {A}_\mathrm {OC_a}\mathrm {OC_a} -\mathcal {A}_\mathrm {OB_a}\mathrm {OB_a} +\mathcal {P}_\mathrm {OB_p}\mathrm {OB_p}\\ &+\mathcal {D}_\mathrm {OC_p}\mathrm {OC_p} +\mathcal {D}_\mathrm {OB_u}\mathrm {OB_u} \end{aligned} \end{aligned}$$

thus clearly identifying the individual contributions of apoptosis, proliferation and differentiation to the overall turnover of the total cell population. For the special case of zero apoptosis, proliferation and differentiation, the total cell population $\textrm{O}$ is trivially conserved.$\square$

In our implementation, the discrete time trajectory of $\textbf{x}$ is algorithmically determined by integrating the nonlinear evolution system $\dot{\textbf{x}}=\textbf{S}(\textbf{x})\textbf{x}+\textbf{f}(\textbf{x};\textbf{c})$ by a Runge–Kutta time integrator over cellular scale time steps $\Delta {\tilde{t}}\ll \Delta {\bar{t}}\equiv \Delta t$ with $\sum \Delta \tilde{t}=\Delta {\bar{t}}\equiv \Delta t$.

*Upscaling:* Bone remodelling changes the size and shape of trabeculae, whereas the overall topology of the trabecular structure remains mostly the same (Mittra et al. 2005). Consequently, increased mechanical stimulation causes trabeculae to become thicker, thereby preserving the number of elements and the connectivity of our trabecular truss network model. In contrast, bone resorption may completely remove individual trabeculae, in the limit even resulting in reduced connectivity of the trabecular truss network. In any case, the net result of these meso scale changes is also an accompanying change in the effective anisotropy.

The surface tissue of trabeculae is assumed to be remodelled by the population of active osteoclasts $\mathrm {OC_a}$ and active osteoblasts $\mathrm {OB_a}$. Depending on the balance between the densities of active osteoclasts $\mathrm {OC_a}$ and active osteoblasts $\mathrm {OB_a}$, the evolution of the cross-sectional area $A^e$ of trabecular element *e* is thus generically defined as

9 $$\begin{aligned} \dot{A}^e = \dot{A}^e\left( \mathrm {OB_a}, \mathrm {OC_a}, \ldots \right) \,. \end{aligned}$$

Here, for the sake of demonstration, we employ the most elementary, linear evolution law for $A^e$ as

10 $$\begin{aligned} \dot{A}^e = f_\textrm{A} \left[ k_{\textrm{form}} \mathrm {OB_a}-k_{\textrm{res}}\mathrm {OC_a}\right] \,, \end{aligned}$$

with $k_{\textrm{res}}$ being the constant resorption rate (volume of bone resorbed per cell per unit time) and $k_{\textrm{form}}$ the constant formation rate (volume of bone formed per cell per unit time). The bone turnover pre-factor $f_\textrm{A}$ (of unit one over length) determines the amount of bone that is turned over due to bone remodelling at the scale of single trabeculae.

**Remark:** In general, $f_\textrm{A}$, rather than being a constant, could be a function dependent on geometrical parameters of the cross section, such as $A^e$ and further biomechanical parameters, i.e. $f_\textrm{A}=f_\textrm{A}(A^e,\ldots )$. However, for the sake of presentation, it is here considered to be constant for all trabeculae in the ZVE. Different, more sophisticated formats for $\dot{A}^e(\mathrm {OB_a}, \mathrm {OC_a}, \ldots )$ will be analysed in a separate contribution. $\square$

Once the cellular problem for the BCPM has been progressed by multiple cellular scale time steps $\Delta {\tilde{t}}$ with $\sum \Delta {\tilde{t}}=\Delta {\bar{t}}\equiv \Delta t$, the increments $\Delta A^e$ are computed by evaluating the right-hand-side of Eq. 10 and multiplying by the macro-meso time step $\Delta {\bar{t}}\equiv \Delta t$.

**Remark:** In our approach, consistent with observation, bone remodelling is characterised by volumetric growth, i.e. by successive accretion of matter with the same material properties as the already existing matter. This translates into the here advocated evolution of trabecular cross sections on the meso scale. A model extension that couples material properties on the meso scale to the various bone cell densities on the cellular scale, if underpinned by experimental evidence, is conceptually straightforward. $\square$

### Algorithmic implementation

**Table 1:** Cellular-Meso-Macro Three-Scale Approach in a Nutshell

Scale	Model	Key Equations	Limitation
Macro	Continuum	$\bar{\varvec{x}} = \bar{\varvec{y}}(\bar{\varvec{X}},{\bar{t}})$	Quasi-Statics
		$$\bar{\varvec{F}} = \overline{\operatorname {Grad}} \bar{\varvec{y}}(\bar{\varvec{X}},{\bar{t}})$$	
		$$\overline{\operatorname {Div}} \bar{\varvec{P}} = \varvec{0}$$	
		$$\bar{\rho }_0=\langle \rho _0\rangle \quad \bar{\varvec{P}}=\langle \varvec{P}\rangle$$	
		$$\bar{\varvec{A}}=\langle \varvec{A}\rangle$$	
Meso	Truss Network	$\varvec{u}_{a\in \mathcal{N}^\textrm{b}} = \bar{\varvec{F}} \cdot \varvec{X}_{a\in \mathcal{N}^\textrm{b}} -\varvec{X}_{a\in \mathcal{N}^\textrm{b}}$	Geometric Fidelity
		$\varvec{f}_a = \varvec{\textsf{A}}_{e=1}^{n_{el}}\,\varvec{n}^e\vert _{a\in \mathcal{N}^\textrm{b}}$	s. Appendix A
		$$\displaystyle \langle \rho _0\rangle = \frac{V_\textrm{T}}{V_\textrm{ZVE}} \, \rho _{0}^\textrm{s}\quad \langle \varvec{P}\rangle = \frac{1}{V_\textrm{ZVE}} \sum _{a\in \mathcal{N}^\textrm{b}} \varvec{f}_a\otimes \varvec{X}_a$$	
		$\displaystyle \langle \varvec{A}\rangle = \frac{1}{V_\textrm{ZVE}} \sum _{a\in \mathcal{N}^\textrm{b}} \sum _{b\in \mathcal{N}^\textrm{b}} \widehat{\varvec{k}}_{ab} \overline{\otimes } \left[ \varvec{X}_a \otimes \varvec{X}_b \right]$	s. Appendix A
		$\dot{A}^e = f_\textrm{A} \left[ k_{\textrm{form}} \mathrm {OB_a}-k_{\textrm{res}}\mathrm {OC_a}\right]$	Constant $f_\textrm{A}$
Cellular	BCPM	$\dot{\textbf{x}}=\textbf{S}(\textbf{x})\textbf{x}+\textbf{f}(\textbf{x}; \textbf{c})$	Stiff ODE System
		$\textbf{x}=[\mathrm {OC_a}, \mathrm {OB_p}, \mathrm {OB_a}]^\textrm{T}$	s. Appendix B

**Fig. 3:**
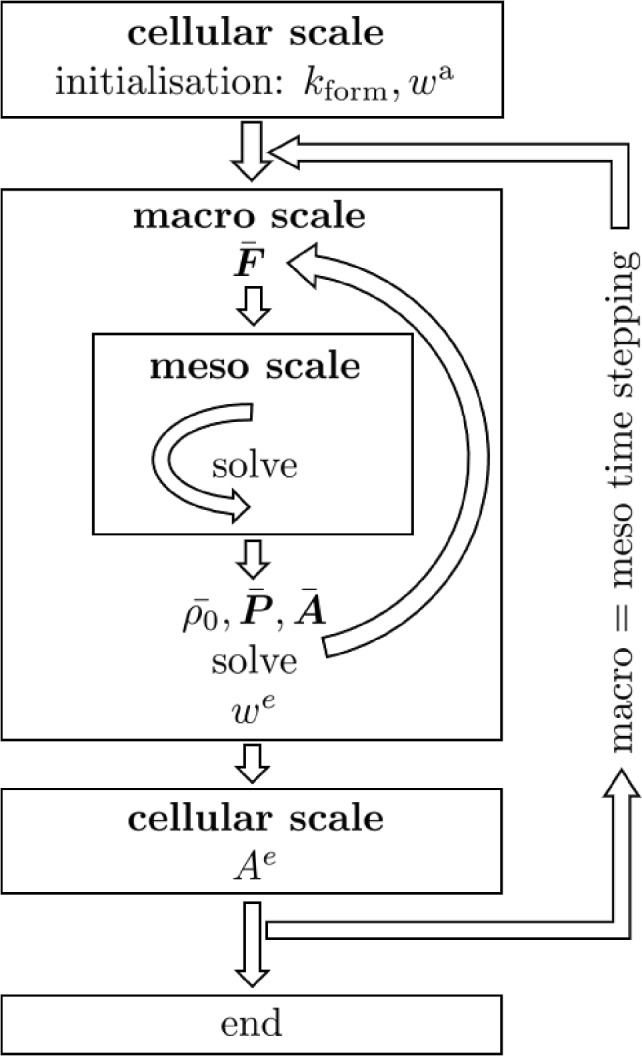
Main steps of the algorithmic implementation: continuum model at the macro scale, trabecular truss network model at the meso scale, and bone cell population model at the cellular scale

The algorithmic implementation is based on the previous set of equations as assembled for convenience in a nutshell in Table 1. To this end, we pursue a staggered solution, where in each macro=meso time step $\Delta {\bar{t}}\equiv \Delta t$ we first evaluate the coupled deformation problem at the macro and meso scales and subsequently evaluate the evolution of the trabecular thickness resulting from the bone cell population model at the cellular scale using cellular scale time steps $\Delta \tilde{t}\ll \Delta {\bar{t}}\equiv \Delta t$. At the macro and meso scales the finite element method is used for the spatial discretisation. In a nutshell, the numerical strategy consists of the following steps, see also Fig. 3:

1. At the macro scale the bone specimen is spatially discretised by finite elements. At each macro scale quadrature point, a discretised ZVE is attached based on the trabecular truss network approach at the meso scale.
2. In a pre-processing step, a steady state calculation is performed to determine the initial parameters of the bone cell population model.
3. The external load is incremented in a macro=meso time step and applied to the spatially discretised specimen at the macro scale.
4. The prescribed boundary displacements of the meso scale ZVE are determined from the macro scale deformation gradient $\bar{\varvec{F}}$ that is computed from the corresponding macro scale nodal displacements.
5. The meso scale deformation problem is solved in an inner loop.
6. The ZVE averaged density, Piola stress and corresponding stiffness tensor $\langle \rho _0\rangle , \langle \varvec{P}\rangle$ and $\langle \varvec{A}\rangle$ are upscaled from the meso to the macro scale.
7. The macro scale deformation problem is solved using a Newton-Raphson iteration scheme in an outer loop involving (4) to (6).
8. The energy storage density $w^e$ in the trabeculae is determined once the coupled macro-meso scale problem has converged. Then, the cross-sectional area of the trabeculae at the meso scale is updated via the bone cell population model driven by the energy storage density $w^e$. Time marching at the cellular scale employs an explicit Runge–Kutta integrator over the cellular time steps.
9. Using the updated deformation at the macro scale and the updated cross-sectional areas at the meso scale as initial conditions, we proceed to the next macro=meso time step.
10. Steps (3) to (9) are repeated until the end of the external load history.

**Remark:** The Bone Cell Population Model (BCPM) is a system of stiff ODEs requiring for its integration a Runge–Kutta time integrator with rather small time steps that resolve a single macro=meso time step of O($10^2$) days by O($10^2$) adaptively chosen cellular time steps. Coupling all three scales monolithically/simultaneously would thus result in prohibitive computational costs when simulating over time spans of O($10^1$) years. Fortunately, staggering/decoupling of the monolithically solved macro/meso scale from the cellular scale comes without severely compromising accuracy, as we analyse and demonstrate in the sequel (3.1.3). $\square$

## Benchmark examples

The performance of the proposed three-scale approach is demonstrated via two benchmark problems. These are here restricted to two space dimensions for the sake of simplicity and demonstration. First, we analyse an elementary (unit square) specimen at the macro scale under uniform uniaxial mechanical load to study in detail the influence of the heterogeneous trabecular truss network at the meso scale and the evolution of pertinent quantities at the cellular scale. Second, to demonstrate the applicability of our approach to non-uniform mechanical loading, we consider an idealised femur head under healthy (baseline BCPM parameters) and osteoporotic (perturbed BCPM parameters) conditions with typical habitual loading at the macro scale stimulating nominal bone density distributions that support the dominant load carrying trajectories. Since in both examples the focus of the study is merely on the qualitative behaviour of the three-scale model, all units are omitted (with the exception for time).

### Uniform benchmark

**Fig. 4:**
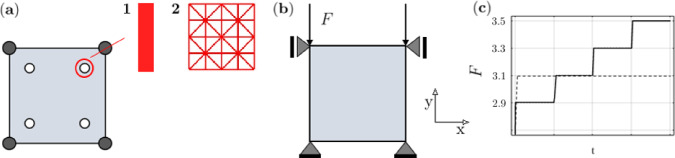
Uniform benchmark: (**a**) one macro scale bilinear finite element with different meso structures representing 1) a single vertical trabecula, and 2) a structured trabecular truss network, (**b**) boundary conditions and mechanical loading at the macro scale with (**c**) step-wise increasing () or constant () forces in vertical direction

This benchmark considers an elementary unit square specimen under compression, which, due to its simplicity, is also used to study the general model behaviour in our recent two-scale approach (Steinmann et al. 2024). We consider this homogeneous example as a benchmark because it has often been considered in the literature for the one-scale approach under uniaxial loading. Therefore, we can compare the results obtained by the novel three-scale approach for exactly the same loading and boundary conditions with other approaches. To this end, a single finite element with bilinear shape functions and $2 \times 2$ Gauss quadrature rule is chosen at the macro scale, see Fig. 4 (panel a). Given that this benchmark problem yields a homogeneous response across all macro scale fields, discretisation with a single finite element is sufficient. To ensure the robustness of our implementation, we verified that various discretisation densities ($1\,\times \,1, 2\,\times \,2, 3\,\times \,3$, etc. bilinear elements) produce, as anticipated, identical evolution of the nominal bone mass density and nominal stress components at the macro scale quadrature points, as well as consistent overall displacements.

The pertinent parameters for the BCPM employed at the cellular scale are given in the appendix (Table 3). The elastic modulus and density of the solid material at the meso scale are set to $E^\textrm{s}= 10000$ and $\rho _{0}^\textrm{s} = 2$, respectively. At the macro scale, the initial value at ${\bar{t}}\equiv t=0$ for the nominal (homogenised) density is set to $\bar{\rho }_{0}^\star = 0.4$. Consequently, at the meso scale the initial value for the cross-sectional area $A_\star ^e$ for each trabecular truss network element *e*, assumed a constant, follows as $A_\star ^e=[\bar{\rho }_{0}^\star /\rho _{0}^\textrm{s}][V_\textrm{ZVE}/L_\textrm{T}]=0.2\,[V_\textrm{ZVE}/L_\textrm{T}]$ with $L_\textrm{T}=\sum _{e=1}^{n_{el}}L_e$ the overall length of the trabecular truss network.

In the sequel, we analyse the two different truss networks in Fig. 4 (panel a1 and a2). These are idealised trabecular architectures that we chose here merely for the sake of demonstration. Their behaviour is studied in detail in 3.1.1$-$3.1.4 and 3.1.6 for the single truss (network) a1 and in 3.1.5 for the multi truss network a2. Earlier, we analysed in detail the influence of various regular and irregular truss network structures at the meso scale (Steinmann et al. 2024). There, we however considered a two-scale approach to cancellous bone remodelling with only phenomenological cross-sectional evolution of the trabeculae. Nevertheless, regarding conclusions with respect to the influence of the meso scale structure on the overall macro scale behaviour we deem the here proposed three-scale approach equivalent to the phenomenological two-scale approach. Therein, the macro scale response including the effective anisotropy depends, as expected, on the meso scale trabecular architecture, indicating that for a realistic computational response the real meso structure needs to be taken into account (refer to the detailed discussion in the second remark in 2.2).

#### Evolution of pertinent quantities at cellular and meso scale

**Fig. 5:**
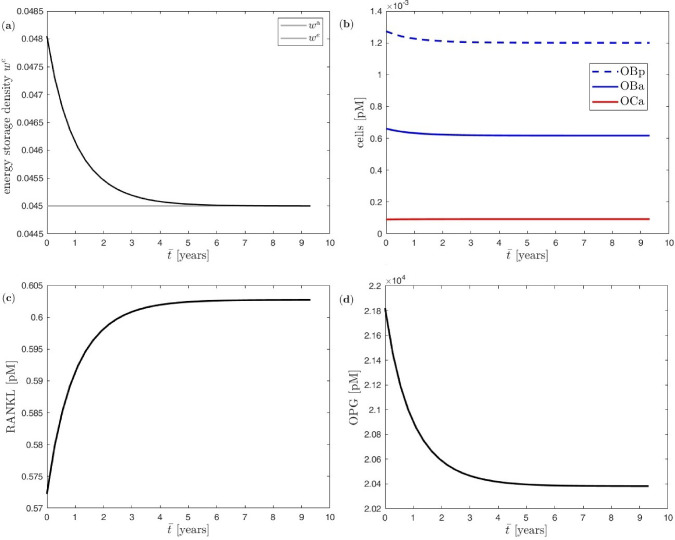
Uniform benchmark example with single trabecula at the meso scale and constant mechanical load at the macro scale: (**a**) evolution of energy storage density, (**b**) evolution of bone cells, (**c**) evolution of RANKL, (**d**) evolution of OPG

Here, first the cellular response to a constant load due to (two) forces of magnitude 3.1 at the macro scale is investigated. The evolution of the system is followed over 35 macro=meso time steps of length $\Delta {\bar{t}} \equiv \Delta t = 100$ days, i.e. over a total time span of $\bar{T}=3500$ days corresponding to 9.6 years. In each macro=meso time step, the Runge–Kutta time integrator chooses a suited number of cellular time steps $\Delta {\tilde{t}}$ so that $\sum \Delta {\tilde{t}}=\Delta {\bar{t}}\equiv \Delta t$ in an automatic and adaptive fashion. The trabecular truss network at the meso scale consists of only a single vertical trabecula, see Fig. 4 (panel a1) with $V_\textrm{ZVE}/L_\textrm{T}=1$ and thus initial cross-sectional area $A^e_\star =0.2$. The initial values for the BCPM are determined in a pre-processing step assuming an initial equilibrium state for a meso scale compressive stress of $-30$. This results in $k_{\textrm{form}}= 52.3179$ and $w^\textrm{a}=30^2/E^\textrm{s}/2=0.045$ as the *attractor value*, i.e. the energy storage density the system drives to in order to attain a new equilibrium state. The bone turnover pre-factor is set to $f_\textrm{A} = 0.005$. With these settings, the evolution of pertinent quantities at the cellular scale result as depicted in Fig. 5.

Initially, the mechanical overloading via (two) forces of magnitude $F=3.1$ induces an increase of the energy storage density $w^e$ in the single trabecula from $w^\textrm{a}=0.045$ to $w^e(t=0)=[2F/A^e_\star ]^2/E^\textrm{s}/2=0.04805$. With the trabecular cross-sectional area increasing over time, $w^e$ asymptotically approaches the attractor value $w^\textrm{a}$ at the end of the loading phase of 9.6 years, see Fig. 5 (panel a).

The resulting changes of precursor and active osteoblasts $\mathrm {OB_p}$ and $\mathrm {OB_a}$ as well as active osteoclasts $\mathrm {OC_a}$ from their respective initial equilibrium values are showcased in Fig. 5 (panel b). Since the mechanical overloading requires new bone formation, the osteoclasts remain essentially unchanged, whereas the precursor and active osteoblasts clearly increase especially in the initial phase of the 9.6 years-long time span.

Since osteoblasts produce RANKL (which promotes differentiation of precursor osteoclasts $\mathrm {OC_p}$ into active osteoclasts $\mathrm {OC_a}$) and OPG (which hinders differentiation of $\mathrm {OC_p}$ into $\mathrm {OC_a}$), changes in osteoblasts also induce changes in RANKL and OPG, see Fig. 5 (panel c+d).

Even though the total simulation time spans over some 9.6 years it appears that all cellular adaptation processes are mostly completed after some 6 years when all cellular quantities approximately reach their new equilibrium values.

**Fig. 6:**
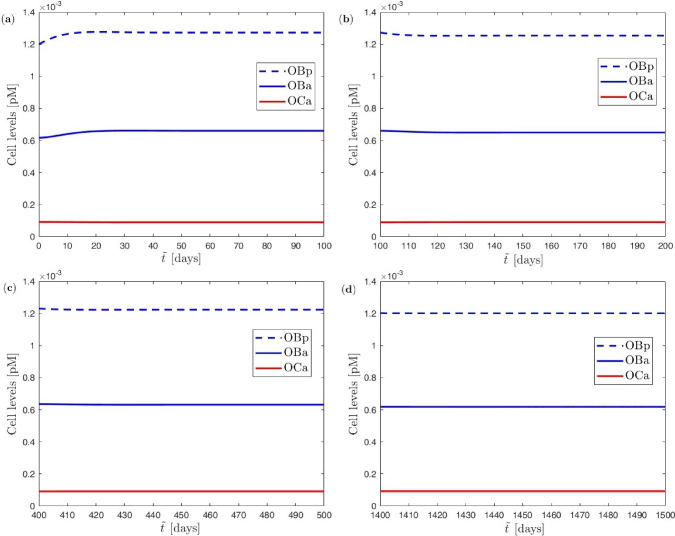
Uniform benchmark example with single trabecula at the meso scale and constant mechanical load at the macro scale: (**a**) cell evolution during first macro=meso time step, (**b**) cell evolution during second macro=meso time step, (**c**) cell evolution during fifth macro=meso time step, (**d**) cell evolution during fifteenth macro=meso time step

Additionally, the evolution of the precursor and active osteoblasts $\mathrm {OB_p}$ and $\mathrm {OB_a}$ as well as active osteoclasts $\mathrm {OC_a}$ as predicted by the BCPM over four specific macro=meso time steps (1st, 2nd, 5th, 15th) with length of 100 days are depicted in Fig. 6. There, the BCPM was evaluated at 189 cellular time steps $\Delta {\tilde{t}}$ as automatically chosen by the Runge–Kutta time integrator. While the active osteoclasts $\mathrm {OC_a}$ remain essentially constant with $\mathrm {OC_a}\approx 9.22 \times 10^{-5}$ over all of the macro-meso time steps due to reasons mentioned above, the precursor and active osteoblasts $\mathrm {OB_p}$ and $\mathrm {OB_a}$ clearly increase initially due to the mechanical overload requiring bone formation before asymptotically returning to their equilibrium value of $\mathrm {OB_p}=1.2 \times 10^{-3}$ and $\mathrm {OB_a}\approx 6.17 \times 10^{-4}$ as defined at the start of the 9.6 year-long simulation phase.

**Fig. 7:**
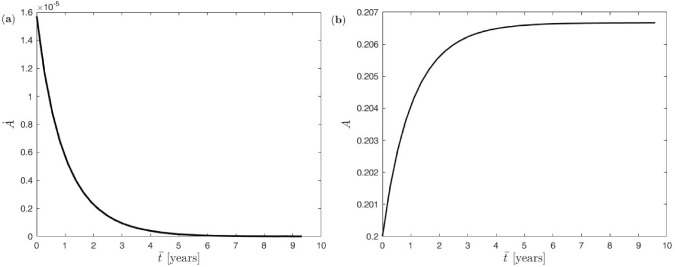
Uniform benchmark example with single trabecula at the meso scale and constant mechanical load at the macro scale: (**a**) evolution of trabecular cross-sectional area rate, (**b**) evolution of trabecular cross-sectional area

The imbalance between bone formation and bone resorption reflects also in the resulting evolution of the cross-sectional area of the single trabecula at the meso scale. While its rate smoothly decreases from its initial positive value to zero, see Fig. 7 (panel a), the cross-sectional area increases until it saturates for the new equilibrium state, see Fig. 7 (panel b). Recall that the rate of the trabecular cross-sectional area in Eq. 10 depends on the formation and resorption rates $k_{\textrm{form}}$ and $k_{\textrm{res}}$ in conjunction with the active osteoblasts and osteoclasts $\mathrm {OB_a}$ and $\mathrm {OC_a}$, respectively. Thus, at equilibrium $k_{\textrm{form}}/k_{\textrm{res}}\equiv \mathrm {OC_a}/\mathrm {OB_a}$ holds (here with $52.3179/350\equiv 0.922/6.17$).

#### Influence of bone turnover pre-factor

**Fig. 8:**
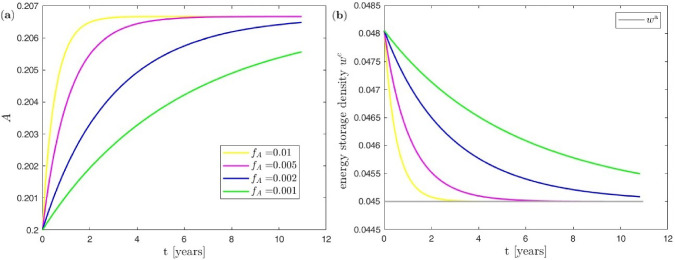
Uniform benchmark example with single trabecula at the meso scale and constant mechanical load at the macro scale: (**a**) effect of bone turnover pre-factor on evolution of trabecular cross-sectional area, (**b**) effect of bone turnover pre-factor on evolution of energy storage density

Here, the effect of the bone turnover pre-factor $f_\textrm{A}$ in Eq. 10 is investigated. For this purpose, the bone turnover pre-factor is varied using again the single trabecula for the truss network at the meso scale. As before, a constant load is applied via (two) forces of magnitude $F=3.1$ over a total simulation time of $\bar{T}=4000$ days (corresponding to some 11 years). The slightly longer total simulation time as compared to the previous subsection with $f_\textrm{A}=0.005$ is here chosen to illustrate the saturating behaviour also for smaller $f_\textrm{A}$. Macro=meso time steps of duration $\Delta {\bar{t}}\equiv \Delta t=$ 50 days are used for time integration. Figure 8 showcases the results for the evolution of the trabecular cross-sectional area and the energy storage density over time.

As a result, the bone turnover pre-factor $f_\textrm{A}$ clearly influences the speed of the remodelling process. The trabecular cross-sectional area increases slower with decreasing $f_\textrm{A}$, and consequently its new equilibrium state is reached later (or not at all in the given simulation time). It shall be noted that here the new equilibrium state for the trabecular cross-sectional area only depends on the mechanical loading and takes the same value independently of $f_\textrm{A}$.

#### Influence of macro=meso time step size

**Fig. 9:**
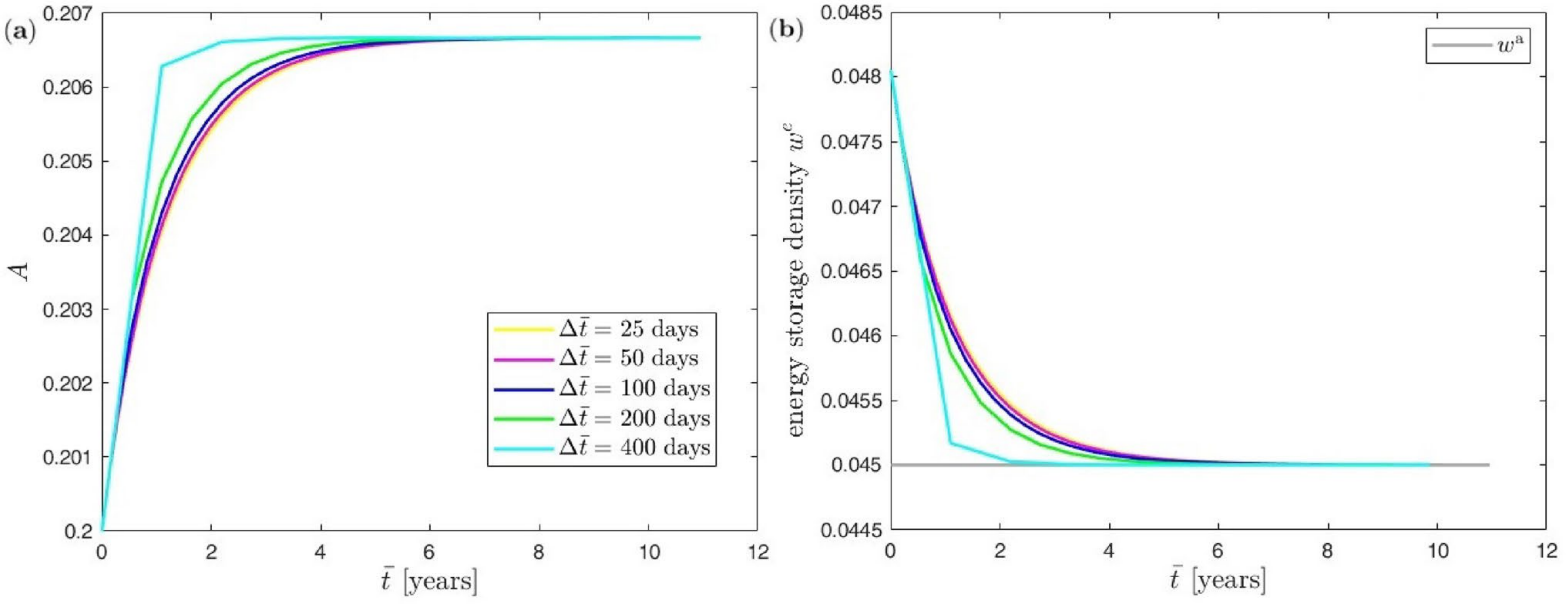
Uniform benchmark example with single trabecula at the meso scale and constant mechanical load at the macro scale: (**a**) effect of macro=meso time step size on evolution of the trabecular cross-sectional area, (**b**) effect of macro=meso time step size on evolution of energy storage density

We do not solve the three-scale problem in an entirely three-way coupled and monolithic fashion, but rather use a staggered algorithmic scheme that first solves the coupled macro=meso problem before solving the cellular problem. Thus, we investigate the effect of the chosen macro=meso time step size on the accuracy for the predicted evolution of the trabecular cross-sectional area and energy storage density. To this end, we again study the truss network with a single trabecula at the meso scale, set $f_\textrm{A}= 0.005$, and apply the constant load phase with (two) forces of magnitude $F=3.1$ at the macro scale when varying the macro=meso time step size (recall that the cellular time step size $\Delta {\tilde{t}}$ is automatically and adaptively chosen by the employed Runge–Kutta time integration scheme). The results are depicted in Fig. 9.

Note that the new equilibrium state for the trabecular cross-sectional area and its energy storage density remains the same for all values of the macro=meso time step size $\Delta \bar{t}\equiv \Delta t$. Thus, the macro=meso time step size merely influences the accuracy of the evolution until saturation is reached, but not the saturation values that are eventually obtained. This may be regarded as underpinning the robustness of our staggered approach. As a result, it appears that, since the formation of bone develops relatively slow, macro=meso time steps of $\Delta t = 100$ days could here be chosen with confidence since sufficient accuracy of the results is maintained.

#### Influence of macroscopic load history

To analyse the influence of the load history at the macro scale, four load phases, each lasting for 3000 days (corresponding to some 8.2 years), with step-wise increasing mechanical loading are applied as schematically depicted in Fig. 4 (panel c). Consequently, the total load history lasts for 12000 days (corresponding to some 32.9 years), whereby a macro=meso time step of $\Delta {\bar{t}} \equiv \Delta t=$ 50 days is used. Starting for the first load phase with (two) forces at the macro scale of magnitude $F=2.9$, the mechanical load is incremented by 0.2 in each subsequent load phase (thus $F=2.9, 3.1, 3.3$ and 3.5 in the four successive load phases).

**Fig. 10:**
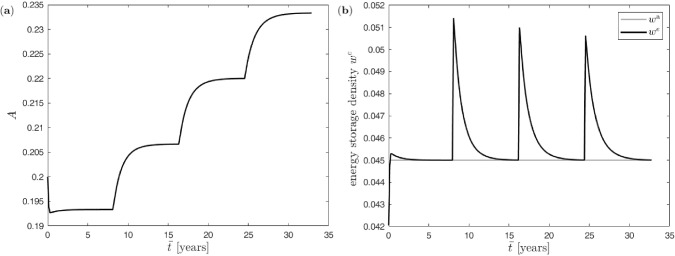
Uniform benchmark example with single trabecula at the meso scale and step-wise mechanical increasing load at the macro scale: (**a**) evolution of trabecular cross-sectional area, (**b**) evolution of the energy storage density

The resulting evolution of the trabecular cross-sectional area and the energy storage density in the single trabecula is shown in Fig. 10. The initial mechanical underloading (with $w^e(t=0)=[2F/A^e_\star ]^2/E^\textrm{s}/2\approx 0.042$) as compared to the attractor value of $w^\textrm{a}=0.045$ results in net bone resorption predominating the entire first loading phase with decreasing trabecular cross-sectional area. Due to the nonlinear dynamics of the three-scale system including the BCPM, an initial undershooting in the trabecular cross-sectional area occurs during the very first time steps. However, the trabecular cross-sectional area eventually recovers and saturates in a new equilibrium state at the end of the first loading phase. Correspondingly, the energy storage density in the trabecula initially increases rapidly from its low starting value $w^e(t=0)$ with a small overshooting followed by a gradual decay to the state of equilibrium as dictated by $w^\textrm{a}$. In the second loading phase, the mechanical stimulation is high enough to induce bone formation. The application of this load phase is accompanied by a clear peak of the energy storage density, which subsequently gradually decreases again to its equilibrium state $w^\textrm{a}$. Similar processes occur in the third and fourth loading phase, whereby the peaks in the energy storage density at the start of each loading phase become smaller due to the already evolved trabecular cross-sectional area. Note that in general, as also engraved in the BCPM (Scheiner et al. 2013; Martin et al. 2019), in case of resorption as in the first loading phase, bone degrades much faster and reaches the saturation state much earlier than in the case of bone formation.

#### Influence of trabecular truss network at meso scale

**Fig. 11:**
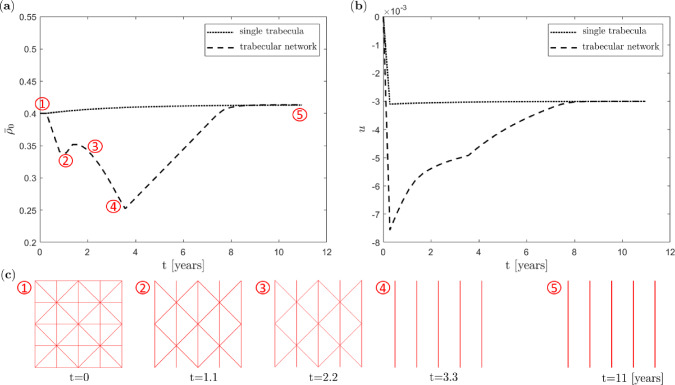
Uniform benchmark example with different trabecular truss network structures at the meso scale and constant mechanical load at the macro scale: (**a**) evolution of (homogenised) nominal density at the macro scale, (**b**) evolution of top nodes’ vertical displacement at macro scale, () single vertical trabecula, () structured trabecular truss network, (**c**) evolution of the structured trabecular truss network at the meso scale over time

In addition, we analyse the influence of the structure of the trabecular truss network at the meso scale. Thus, in addition to the previously studied single trabecula structure for the truss network in Fig. 4 (panel a1), we now also analyse a *structured truss network* with 25 uniformly distributed nodes connected by 56 trabeculae, see Fig. 4 (panel a2). Again, a constant load by (two) forces of magnitude $F=3.1$ is applied over $\bar{T}=4000$ days (corresponding to some 11 years) at the macro scale. Here, to better control the speed of the remodelling process, the bone turnover pre-factor is set to $f_\textrm{A}=0.002$. For this example, the (homogenised) nominal density $\bar{\rho }_0$ at the macro scale as determined from Eq. 7 is monitored in order to analyse the effect of the different trabecular truss network structures at the meso scale. The corresponding results as well as the evolution of the displacement are displayed in Fig. 11.

Observe that in the case of a *single trabecula*, the (homogenised) nominal density $\bar{\rho }_0$ as well as the top node’s vertical displacement *u* evolve monotonously at the macro scale in Fig. 11 until the new equilibrium state is reached.

In contrast, the (homogenised) nominal density $\bar{\rho }_0$ at the macro scale for the *structured trabecular truss network* in Fig. 11 (panel a) first decreases quickly, then increases more slowly and decreases again. Finally, $\bar{\rho }_0$ increases again until it approaches the saturation state. Looking closer at the evolution of the truss network at the meso scale, see Fig. 11 (panels c1-c5), it is apparent that the first decrease in $\bar{\rho }_0$ is related to the rapid resorption of the horizontal trabeculae that are oriented perpendicular to the load direction, see Fig. 11 (panels c2,c3). The second decrease in $\bar{\rho }_0$ stems from the subsequent resorption of the diagonally arranged trabeculae, see Fig. 11 (panel c4), whereas only the vertical trabeculae steadily thicken until saturation is finally reached, see Fig. 11 (panel c5). Eventually, only the vertical trabeculae persist, thus revealing the ability of the advocated three-scale approach to capture the anisotropic evolution of bone meso architecture. In particular, our approach captures the loss of trabeculae, respectively the permanent thinning of the cancellous bone as a hallmark of osteoporosis.

It is finally interesting to observe in Fig. 11 (panel b) that the top node’s vertical displacement *u* at the macro scale approaches the same equilibrium value for the single trabecula and the structured trabecular truss network.

The effect of unstructured trabecular networks has been investigated within our recent two-scale approach (Steinmann et al. 2024). Corresponding analyses are not repeated here for the sake of conciseness.

#### Influence of external RANKL injection and anabolic feedback

**Fig. 12:**
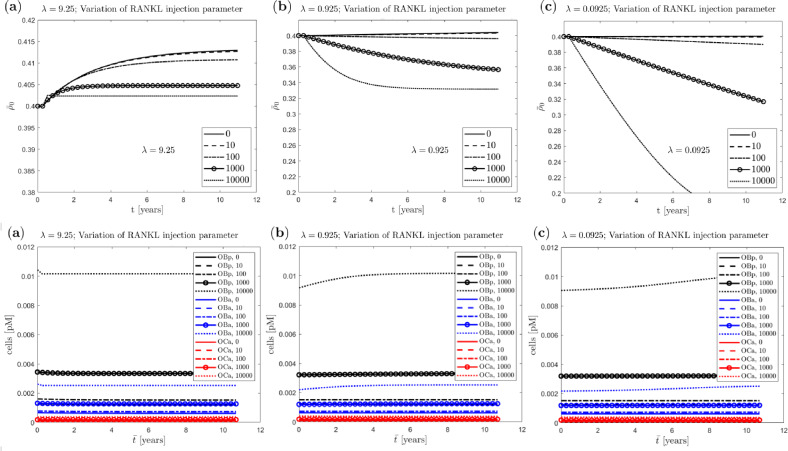
Uniform benchmark example with single trabecula at the meso scale and constant mechanical load at the macro scale: evolution of (homogenised) nominal density at the macro scale (top row) and corresponding cell numbers (bottom row) with variation of the RANKL injection parameter and different anabolic strength parameters (**a**) $\lambda =9.25$, (**b**) $\lambda =0.925$, (**c**) $$\lambda =0.0925\ $$

Finally, to demonstrate the advantage of the new multi-scale approach allowing to couple macroscopic mechanical loading and the bone cell population model, we investigate the influence of an external RANKL injection, which mimics induction of osteoporosis, together with the anabolic strength parameter $\lambda$ of the mechanostat model. Again we apply the loading in a single step and use the single trabecula structure for the truss network. We vary $\lambda$ between 9.25, 0.925 and 0.0925 and the RANKL injection parameter $\textrm{RANKL}_d$ between 0, 10, 100, 1000 and 10000. This gives us a total of 15 pairings, for each of which the resulting density evolution as well as the corresponding evolution of cell numbers are shown in Fig. 12. Note that $\lambda =9.25$ and $\textrm{RANKL}_d=0$ represent the baseline parameters.

In the case of large anabolic strength parameter $\lambda$, the (homogenised) nominal density $\bar{\rho }_0$ at the macro scale increases rapidly with different saturation states depending on $\textrm{RANKL}_d$ (panel a, top). For $\lambda$ one order of magnitude smaller, minimal density increase can still be observed for small $\textrm{RANKL}_d$, but for larger $\textrm{RANKL}_d$ the density decreases steadily with a tendency towards saturation (panel b, top). In case of the smallest $\lambda$, a marginal increase in density can only be observed for $\textrm{RANKL}_d$ equal to 0, otherwise the density decreases, partly dramatically, with larger $\textrm{RANKL}_d$ (panel c, top).

**Fig. 13:**
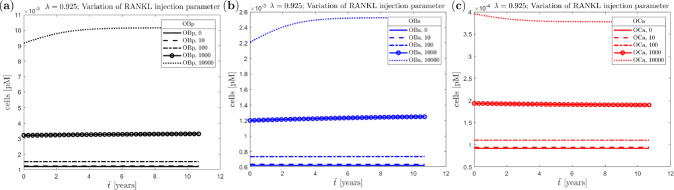
Uniform benchmark example with single trabecula at the meso scale and constant mechanical load at the macro scale for $\lambda =0.925$ (detail of Fig. 12 bottom (**b**)): cell numbers with variation $\textrm{RANKL}_d$ (**a**) precursor osteoblasts, (**b**) active osteoblasts, (**c**) active osteoclasts

The dependence of the cell numbers on the parameter $\lambda$ is reflected by a very small increase, which is not easily recognisable in Fig. 12. Other than that, there is a more perceptible influence of the parameter $\textrm{RANKL}_d$, which is directly incorporated into the cell population model, in that higher values result in a larger number of cells. This observation is consistent with the literature. The cell numbers for $\lambda =0.925$ and the variation of the RANKL injection parameter $\textrm{RANKL}_d$ are shown in more detail in Fig. 13, corresponding to Fig. 12 (**b**), bottom.

### Proximal femur head in healthy and osteoporotic conditions

**Fig. 14:**
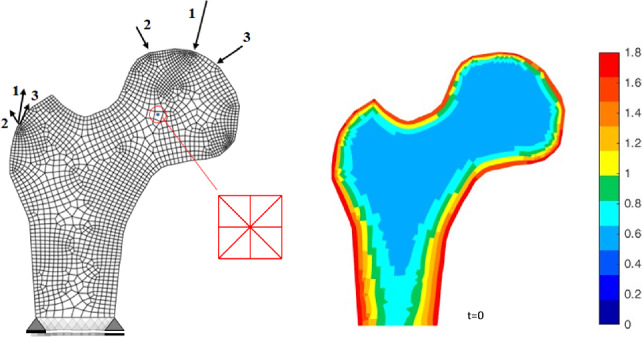
Proximal femur head: macro scale boundary conditions and finite element mesh (left) and the initial (effective) nominal density distribution (right). The cortical bone, i.e. the regions coloured from red to yellow, is modelled by a one-scale approach, the trabecular bone, i.e. the regions coloured from green to blue, is modelled by the novel three-scale approach. For the latter, the structured ZVE as displayed (middle) is considered for demonstration purposes. Note, however, that this is a strongly idealised ZVE since the cancellous network in real bone is not uniformly distributed

In this study, we apply the proposed three-scale approach to a simplified biomechanical problem by qualitatively analysing a two-dimensional section of a proximal femur head. The macro scale finite element mesh comprises 1988 bilinear continuum elements with $2 \times 2$ Gauss quadrature and 2124 node points, as depicted in Fig. 14 (left) and also described in Steinmann et al. (2024). The consistent linearisation of the averaged Piola stress ensures quadratic rate of convergence for the incremental-iterative Newton scheme, requiring only a few iterations per load increment. Thanks to the stringent geometric assumptions regarding the underlying symmetric and structured trabecular network, similar to the one used in the previous examples, but with only three nodes per direction, see Fig. 14 (middle), the computational load of the proposed three-scale approach remains manageable.

The habitual daily loading at the femur head can be characterised by three representative load cases corresponding to the mid-stance, extreme abduction, and extreme adduction phases of the gait cycle. For this study, accurate determination of the magnitude and spatio-temporal distribution of habitual daily loading as described in Christen et al. (2013) is beyond our scope. Instead, we simplify macro scale mechanical loading by simultaneously applying the loads detailed in Carter and Beaupré (2001) as single forces at the corresponding node points of the macro scale discretisation. Referring to Fig. 14 pairs of single forces representing (1) the mid-stance phase of gait (2317 $\text {N/+24}^\circ$ and 703 $\text {N/+28}^\circ$), (2) the extreme range of abduction (1158 $\text {N/-15}^\circ$ and 351 $\text {N/-8}^\circ$), and (3) the extreme range of adduction (1548 $\text {N/+56}^\circ$ and 468 $\text {N/+35}^\circ$) are applied simultaneously. It is evident that variations in the mechanical load in terms of magnitude, direction, distribution, and location critically affect the resulting bone density distribution. Therefore, the following results are qualitative and aim to demonstrate the overall applicability, performance, and behaviour of the proposed three-scale approach.

The phenomenological one-scale approach described in Schmidt et al. (2021) serves for the treatment of the outer (dense) cortical bone layer. The initial nominal density at the outer cortical bone layer is set to $\bar{\rho }_{0}^\star = 1.8$ and to $\bar{\rho }_{0}^\star = 0.55$ for the inner cancellous bone. The transition zone between cortical bone, including the regions coloured from red to yellow in Fig. 14 (right), and cancellous bone, including the regions coloured from green to blue in Fig. 14 (right), is described by a *sigmoidal* function. For the meso scale, only macro scale regions with an initial nominal density smaller than $\bar{\rho }_{0}^\star = 1$, i.e. only the regions coloured green to blue in Fig. 14 (right) are assigned a ZVE. For these, for demonstration purposes, we consider a simple structured meso scale ZVE, depicted in Fig. 14 (middle). For examples of alternative, more complex, also unstructured meso scale ZVEs, please refer to Steinmann et al. (2024). The evolution of the individual trabecular thicknesses is determined via the cell population model at the cellular scale. Macro scale regions with relatively high initial target density representing cortical bone are captured using the one-scale approach, while macro scale regions representing cancellous bone are described using the novel three-scale approach. The set of pertinent parameters for simulating the proximal femur with the one-scale and the novel three-scale approach are listed in the appendix.

**Fig. 15:**
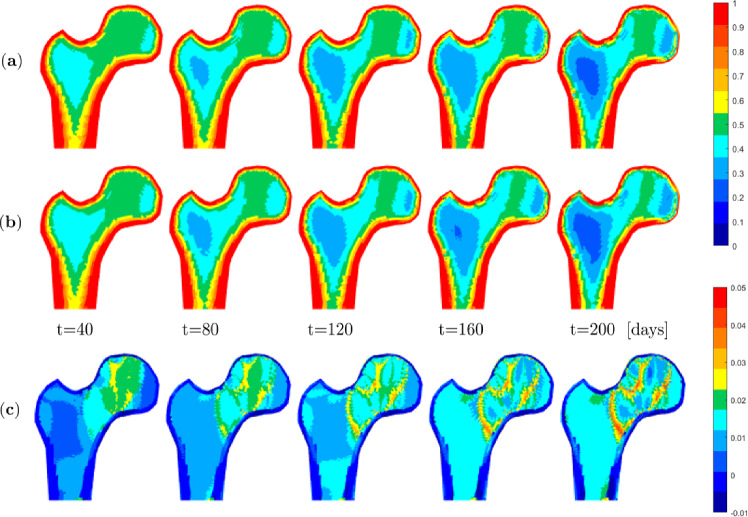
Proximal femur head: (effective) nominal density at different time points using the three-scale approach using a symmetric mesoscale structure with three nodes per direction: baseline parameters (**a**), osteoporotic (**b**), and difference between both (**c**)

Figure 15 illustrates the development of the (effective) nominal density distribution for certain simulation time points over a total period of 200 days. As a baseline, we use the anabolic strength parameter of the mechanostat model set to $\lambda =9.25$ pM $\hbox {day}^{-1}$ and the parameter of the external RANKL injection $\textrm{RANKL}_d$ set to zero (see upper row (**a**)). However, in order to simulate an osteoporotic bone, we assume that the mechanostat weakens with increasing age and that the slope of the anabolic path becomes flatter as a result, i.e. $\lambda =0.925$ pM $\hbox {day}^{-1}$, and the parameter of the external RANKL injection increases, in this example to $\textrm{RANKL}_d=1000$ pM $\hbox {day}^{-1}$ (see middle row (**b**)).

Essentially, the (effective) nominal density distribution is in both cases qualitatively similar to that of the one- and two-scale models reported in Steinmann et al. (2024). Thereby, as a general trend, all simulations consistently predict a slight relative increase in (effective) nominal density in the highly loaded zone on the medial side and a lower bone density in the femoral neck region, with also the dense cortical bone shell at the outer layer of the femoral head evolving qualitatively similar in all simulations.

However, the three-scale approach can additionally reveal much richer detail and also allows capturing pathological cases. In particular, the differences in the density evolution between the baseline healthy and the osteoporotic bone, i.e. the influence of the various parameters, are shown by the difference in the corresponding element values in the last row (**c**) in Fig. 15. As expected, the osteoporotic bone has a slightly reduced density in the entire interior. The most significant differences occur at the peripheries of the more highly loaded zone of the femoral head. In the case of osteoporosis, the density in this area does not develop accordingly due to the weaker mechanical response and the increased activity of the osteoclasts.

Note that this example is intended solely to demonstrate the effectivity of our novel three-scale approach. We have conducted preliminary studies on various meso structures (regarding the number of nodes and trabeculae, as well as their overall structure) and in-depth parameter studies (particularly of the cell population model). However, these would unnecessarily exceed the scope of this publication. Nevertheless, this simple example already demonstrates the potential of the three-scale approach to predict the development of the (effective) nominal density based on mechanobiological processes at the cellular scale. Additionally, it allows for a virtual zoom-in into the spongy bone, enabling the observation of the, potentially anisotropic evolution of the trabecular architecture within individual ZVEs.

## Discussion and conclusion

We proposed a novel three-scale trabecular bone remodelling approach taking into account the mechanically loaded macro (organ) scale, the mechanobiologically adaptive trabecular meso (tissue) scale and the cellular (biological process) scale. We elucidated the role of some key modelling parameters when applied to a set of benchmark problems. As a demonstration of its efficacy, we finally applied the novel three-scale approach to the celebrated Carter-Beaupré 2D femur head example, thereby analysing and comparing bone remodelling in a healthy subject and under osteoporotic conditions.

The innovation of our three-scale approach lies in the detailed consideration of the osteoclastic and osteoblastic cell activity that drives the bone remodelling process of the trabecular architecture on the meso scale, which in turn is upscaled to the macro (organ) scale. This approach is conceptually different to two-scale bone adaptation approaches where the BCPM is formulated typically for a representative volume element only (Lerebours et al. 2016). The latter models only allow for predictions of porosity (bone volume fractions), but no monitoring of changes in bone meso scale architecture and resulting effective anisotropy.

Thus, the simultaneous consideration of changes in the porosity/bone volume fraction, and in the meso structure, at the ZVE scale represents another significant advantage of the current three-scale approach compared to established two-scale models of bone adaptation. This is particular relevant for trabecular bone adaptation which may be due to bone disease, drug treatments and/or physical exercise leading to changes in the trabecular meso architecture. For example, the transition of an initial isotropic bone meso structure into an anisotropic meso structure is automatically and explicitly captured by our approach via the cross-sectional changes (including the limiting case of complete resorption) of individual trabecula. Phenomenological anisotropic bone remodelling formulations were earlier suggested e.g. by Jacobs et al. (1997), Martin et al. (2020). However, different to ours, these phenomenological approaches lack mechanobiological foundation and explicit spatio-temporal resolution of the trabecular meso architecture.

We find that in our approach, the type and degree of anisotropy captured depends intimately on the chosen initial ZVE truss network architecture. Optimally, the ZVE architecture follows directly from an elaborate imaging-discretisation pipeline. Notably, though, for the considered benchmark problems considering only simple, structured ZVE truss networks, the change in effective anisotropy for the final adapted state is already notable.

We also demonstrate that our proposed three-scale approach is compatible with established one- and two-scale approaches regarding the nominal macro scale density evolution when applied to the classical Carter-Beaupré femur head example. However, in stark contrast to the established approaches, the three-scale approach allows additionally for explicit analysis of the rich detail in structural and cellular processes at the meso and cellular scales.

Furthermore, we show that the three-scale model enables simulation of bone adaptation under disease conditions, here exemplified (for the sake of demonstration) for the case of an osteoporotic femur head of Carter-Beaupré-type. This is straightforwardly achieved by activating the external RANKL dosage through the corresponding injection port of the BCPM. Recall that the BCPM potentially also allows activation of a number of other dosage ports (OPG, PTH, RANK, $\textrm{TGF}_\beta$) so as to simulate drug treatment and/or diseases. Clearly, it would not be possible simulating clinically relevant conditions with purely mechanically based bone adaptation approaches. Our simulation reveal that the combined perturbations in the anabolic and catabolic pathways as captured by reduction of the anabolic strength parameter $\lambda$ and activation of the $\textrm{RANKL}_d$ injection effectively predict changes in bone under osteoporotic conditions.

There are of course still limitations: Here, to reduce computational costs, we restricted our simulations to two-dimensional benchmark problems. The technicalities coming with an extension to three dimensions are conceptionally straightforward, though tedious, and are on our agenda for the near future. This will then enable an increase in the complexity and thus realism of the trabecular architecture that we plan to generate from an elaborate imaging-discretisation pipeline. Thereby, from the structural mechanics viewpoint, discretisation of the intricate trabecular meso structure requires, besides the current truss elements, incorporation and coupling of the entire bandwidth of structural (bars/beams, membranes/shells) and bulk finite elements. This, however, constitutes rather a computational mechanics than a mechanobiological challenge. Overall, computational efficiency was not yet central in this contribution that aims in first creating a viable three-scale, cellular-meso-macro approach. Our immediate future work targets the reduction of computational cost^3^ and an increase in geometric complexity and realism of the approach.

Summarising, our novel three-scale approach to trabecular bone remodelling provides an effective framework and suited launch pad for future full-scale, high-realism bone adaption simulations that allow for patient-specificity regarding the prediction and drug treatment of bone loss with age and/or under deregulatory circumstances.

## Figures and Tables

**Fig. 16:**
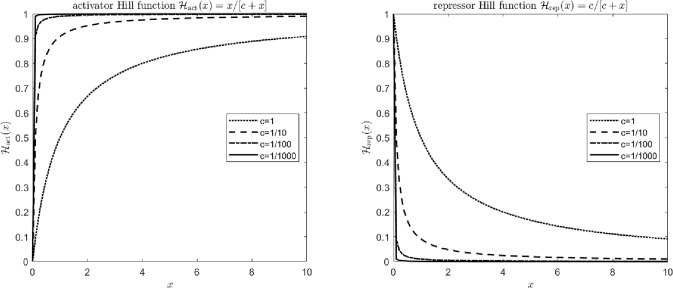
Hill activator (left) and repressor (right) functions

## Meso scale network model

Here, further technical details pertaining to the network model at the meso scale are given:

*Notation:* For two first-order tensors (vectors) $\varvec{a}\in \mathbb {R}^d$ and $\varvec{b}\in \mathbb {R}^d$ the notation $[\varvec{a}\backslash \varvec{b}]\in \mathbb {R}^{2d}$ refers to a first-order tensor (vector) composed from $\varvec{a}$ and $\varvec{b}$. For two second-order tensors $\varvec{A}\in \mathbb {R}^d\times \mathbb {R}^d$ and $\varvec{B}\in \mathbb {R}^d\times \mathbb {R}^d$ the notation $[\varvec{A}|\varvec{B}]\in \mathbb {R}^d\times \mathbb {R}^{2d}$ refers to a tensorial object composed from $\varvec{A}$ and $\varvec{B}$ and mapping from $\mathbb {R}^{2d}$ to $\mathbb {R}^d$. The special dyadic product $\overline{\otimes }$ of two second-order tensors is defined as $\left[ \varvec{a} \, \overline{\otimes } \,\varvec{B} \right] _{iJkL}:= a_{ik} \, B_{JL}$.

*Kinematics:* A truss network consists of a set of node points $\mathcal{N}$ (globally) numbered by $a=1,\ldots n_{np}$ connected by elements numbered by $e=1,\ldots n_{el}$. The coordinates and displacements of the node points are $\varvec{X}_a$ and $\varvec{u}_a$, respectively, for all $a=1,\ldots n_{np}$. The assignment of node points to elements is indicated via a connectivity list that reads $c^e=[a,b]$ for all $e=1,\ldots n_{el}$ and $a,b\in \mathcal{N}$. Then, the element vectors of coordinates and displacements follow as $\varvec{X}^e=[\varvec{X}_a\backslash \varvec{X}_b]$ and $\varvec{u}^e=[\varvec{u}_a\backslash \varvec{u}_b]$, respectively.

We introduce the projection $\varvec{J}=[-\varvec{I}\;|+\varvec{I}]$ with $\varvec{I}$ the second-order unit tensor. Then, the element length and director (a unit vector) follow as $L^e=\vert \varvec{J}\cdot \varvec{X}^e\vert$ and $\varvec{d}^e=\varvec{J}\cdot \varvec{X}^e/L^e$, respectively (note that $\varvec{J}\cdot \varvec{X}^e=\varvec{X}_b-\varvec{X}_a$). The strain in an element is constant and reads $\epsilon ^e=\varvec{b}^e\cdot \varvec{u}^e$ with the strain–displacement operator $\varvec{b}^e=\varvec{d}^e\cdot \varvec{J}/L^e$ (note that $\varvec{d}^e\cdot \varvec{J}=[-\varvec{d}^e\;\backslash +\varvec{d}^e]$, thus $\epsilon ^e=\varvec{d}^e\cdot [\varvec{u}_b-\varvec{u}_a]/L^e$ is the elongation of an element divided by its original length).

*Statics:* The derivative of the total energy storage $W^e=E^\textrm{s}A^eL^e[\epsilon ^e]^2/2$ with respect to the element vector of displacements $\varvec{u}^e$ renders the element vector of internal forces $\varvec{n}^e=\partial W^e/\partial \varvec{u}^e=n^eL^e\varvec{b}^e$ with the constant normal force $n^e=E^\textrm{s}A^e\epsilon ^e$ (note that $\varvec{n}^e=n^e[-\varvec{d}^e\;\backslash +\varvec{d}^e]$). The element stiffness tensor follows furthermore from linearisation as $\varvec{k}^e=\partial \varvec{n}^e/\partial \varvec{u}^e=k^e[L^e]^2\varvec{b}^e\otimes \varvec{b}^e$ with the extensional stiffness $k^e=E^\textrm{s}A^e/L^e$ (note that $\varvec{k}^e=k^e[-\varvec{d}^e\;\backslash +\varvec{d}^e]\otimes [-\varvec{d}^e\;\backslash +\varvec{d}^e]$). Finally, (in the absence of external forces) the assembly of all element contributions results in the global residual $\varvec{r}=-\bigcup _e \varvec{n}^e\doteq \varvec{0}$ and its linearisation $\varvec{k}\doteq \bigcup _e \varvec{k}^e$ so that the (Newton) update for the global vector of displacements $\varvec{u}$ reads $\varvec{u}\rightarrow \varvec{u}+\varvec{k}^{-1}\cdot \varvec{r}$.

*Upscaling:* The fourth-order stiffness tensor $\langle \varvec{A}\rangle$ reads as

$$\begin{aligned} \langle \varvec{A}\rangle = \frac{1}{V_\textrm{ZVE}} \sum _{a\in \mathcal{N}^\textrm{b}} \sum _{b\in \mathcal{N}^\textrm{b}} \widehat{\varvec{k}}_{ab} \overline{\otimes } \left[ \varvec{X}_a \otimes \varvec{X}_b \right] \,. \end{aligned}$$

Here, $\widehat{\varvec{k}}_{ab}$ denote second-order tensorial elements (associated with node points $a, b\in \mathcal{N}^\textrm{b}$ of the ZVE) of the condensed ZVE stiffness $\widehat{\varvec{k}}$ which follows from the partitioning of the total ZVE stiffness $\varvec{k}$ into prescribed displacements at the ZVE boundary (superscript $\textrm{p}$) and the retained displacements within the ZVE (superscript $\textrm{r}$)

$$\begin{aligned} \widehat{\varvec{k}} = \varvec{k}^\textrm{pp} - \varvec{k}^\textrm{pr}\cdot [\varvec{k}^\textrm{rr}]^{-1}\cdot \varvec{k}^\textrm{rp} \,. \end{aligned}$$

## Cellular scale BCPM

Here, further details of the BCPM (bone cell population model) employed (Scheiner et al. 2013) at the cellular scale are given:

Differentiation and apoptosis of cells are regulated via Hill *activator* and *repressor* functions that read generically as

$$\begin{aligned} \mathcal {H}_\textrm{act}(x)=x/[c+x], \quad \text{ and }\quad \mathcal {H}_\textrm{rep}(x)=c/[c+x] \end{aligned}$$

respectively, with *x* the variable and *c* a constant. Typical Hill activator and repressor functions for different values of $c=[1, 1/10, 1/100, 1/1000]$ are showcased in Fig. 16.

Taken together, we set

$$\begin{aligned} \mathcal {D}_\mathrm {OB_p}= & D_\mathrm {OB_p}\bar{\mathcal {D}}_\mathrm {rep,OB_p}\\ \mathcal {D}_\mathrm {OB_u}= & D_\mathrm {OB_u}\bar{\mathcal {D}}_\mathrm {act,OB_u}\\ \mathcal {A}_\mathrm {OC_a}= & A_\mathrm {OC_a}\bar{\mathcal {A}}_\mathrm {act,OC_a} \end{aligned}$$

with $D_\mathrm {OB_p}$, $D_\mathrm {OB_u}$, and $A_\mathrm {OC_a}$ maximum differentiation and apoptosis rates, respectively, that serve as parameters of the BCPM, and $\bar{\mathcal {D}}_\mathrm {rep,OB_p}$, $\bar{\mathcal {D}}_\mathrm {act,OB_u}$, and $\bar{\mathcal {A}}_\mathrm {act,OC_a}$ the corresponding repressor and activator Hill-type functionals. These depend on the concentration of the growth factor $\textrm{TGF}_\beta$ and corresponding parameters $K_\mathrm {OB_p}$, $K_\mathrm {OB_u}$, and $K_\mathrm {OC_a}$, respectively. The growth factor $\textrm{TGF}_\beta$ in turn is a linear function of the active osteoclasts $\mathrm {OC_a}$, i.e. $\textrm{TGF}_\beta =[R_\mathrm {TGF_\beta }\mathrm {OC_a}+S_\mathrm {TGF_\beta }]/D_\mathrm {TGF_\beta }$ with $R_\mathrm {TGF_\beta }$, $S_\mathrm {TGF_\beta }$, and $D_\mathrm {TGF_\beta }$ further parameters (here set so that $\textrm{TGF}_\beta =\mathrm {OC_a}$). Finally, $\mathcal {A}_\mathrm {OB_a}=A_\mathrm {OB_a}$ is the constant apoptosis rate for the $\mathrm {OB_a}$ pool, another parameter of the BCPM. In summary, we thus have that:

- Differentiation of precursor osteoblasts $\mathrm {OB_p}$ into active osteoblasts $\mathrm {OB_a}$ is *negatively* regulated via the *repressor* functional $\mathcal {D}_\mathrm {OB_p}=\mathcal {D}_\mathrm {OB_p}\left( \textrm{TGF}_\beta (\mathrm {OC_a})\right)$.
- Differentiation of uncommitted osteoblasts $\mathrm {OB_u}$ into precursor osteoblasts $\mathrm {OB_p}$ is *positively* regulated via the *activator* functional $\mathcal {D}_\mathrm {OB_u}\left( \textrm{TGF}_\beta (\mathrm {OC_a})\right)$.
- Apoptosis of active osteoclasts $\mathrm {OC_a}$ is *positively* regulated via the *activator* functional $\mathcal {A}_\mathrm {OC_a}=\mathcal {A}_\mathrm {OC_a}\left( \textrm{TGF}_\beta (\mathrm {OC_a})\right)$.
- Apoptosis of active osteoblasts $\mathrm {OB_a}$ is captured via the constant apoptosis rate $\mathcal {A}_\mathrm {OB_a}=A_\mathrm {OB_a}$.

Collectively, $\mathcal {D}_\mathrm {OB_p}$, $\mathcal {D}_\mathrm {OB_u}$, $\mathcal {A}_\mathrm {OC_a}$, and $\mathcal {A}_\mathrm {OB_a}$ are parameterised by ten constants. These are in addition to the numerous constants that parameterise $\mathcal {P}_\mathrm {OB_p}$ and $\mathcal {D}_\mathrm {OC_p}$ as discussed in the main text. The pertinent relations of the here employed BCPM due to Scheiner et al. (2013) are assembled in detail for convenience in Table 2. The corresponding model parameters used in this contribution are listed in Table 3. Taken together, the parameters employed in the BCPM are a result of careful calibration in the extensive series of analyses in the previous contributions of Pivonka and collaborators (2008–2020), thereby wherever possible resorting to experimental evidence.

**Table 2:** Summary of the BCPM

Function		Expression
$\mathcal {D}_\mathrm {OB_p}$	=	$\displaystyle D_\mathrm {OB_p}\frac{K_\mathrm {OB_p}}{K_\mathrm {OB_p}+\textrm{TGF}_\beta }$
$\mathcal {D}_\mathrm {OB_u}$	=	$\displaystyle D_\mathrm {OB_u}\frac{\textrm{TGF}_\beta }{K_\mathrm {OB_u}+\textrm{TGF}_\beta }$
$\mathcal {A}_\mathrm {OC_a}$	=	$\displaystyle A_\mathrm {OC_a}\frac{\textrm{TGF}_\beta }{K_\mathrm {OC_a}+\textrm{TGF}_\beta }$
$\textrm{TGF}_\beta$	=	$\mathrm {OC_a}$
$\mathcal {A}_\mathrm {OB_a}$	=	$A_\mathrm {OB_a}$
$\mathcal {P}_\mathrm {OB_p}$	=	$\displaystyle P_\mathrm {OB_p}\Big [\frac{1}{2}+\frac{\lambda }{2}[{\bar{w}} ^e-1]\Big ]\in \Big [\frac{1}{2}, 1\Big ]$
$\mathcal {D}_\mathrm {OC_p}$	=	$\displaystyle D_\mathrm {OC_p}\frac{\mathrm {RANKL_{R}}}{K_\mathrm {RANKL_R}^{\mathrm d}+\mathrm {RANKL_{R}}}$
$\mathrm {RANKL_{R}}$	=	$K_\mathrm {RANKL_R}^{\mathrm a}\textrm{RANK}\,\textrm{RANKL}$
$\displaystyle \frac{\textrm{RANKL}\, \delta }{\mathrm {RANKL^m}}$	=	$\displaystyle \frac{\beta _\textrm{RANKL}+\mathcal {P}_\textrm{RANKL}}{\beta _\textrm{RANKL}+D_\textrm{RANKL} \mathrm {RANKL^m}}$
$\mathrm {RANKL^m}$	=	$\displaystyle N_\mathrm {OB_p}\mathrm {OB_p}\frac{\textrm{PTH}}{K_\textrm{PTH}^{\mathrm a}+\textrm{PTH}}$
$\delta -1$	=	$K_\mathrm {RANKL_O}^{\mathrm a}\textrm{OPG}+K_\mathrm {RANKL_R}^{\mathrm a}\textrm{RANK}$
$\mathcal {P}_\textrm{RANKL}$	=	$\kappa \max (0,1-{\bar{w}}^e)+\mathrm {RANKL_d}$
$\textrm{PTH}$	=	$\displaystyle \frac{\beta _\textrm{PTH}}{D_\textrm{PTH}}$
$\displaystyle \frac{\textrm{OPG}\;\;\,}{\mathrm {OPG^m}}$	=	$\displaystyle \frac{\beta _\textrm{OPG}}{\beta _\textrm{OPG}+D_\textrm{OPG}\mathrm {OPG^m}}$
$\beta _\textrm{OPG}$	=	$\displaystyle B_\textrm{OPG}\mathrm {OB_a}\frac{K_\textrm{PTH}^{\mathrm r}}{K_\textrm{PTH}^{\mathrm r}+\textrm{PTH}}$

**Table 3:** Pertinent Parameters of the BCPM

Parameter	Numerical Value	Unit
$A_\mathrm {OC_a}$	$5.6487 \times 10^0$	$\textrm{day}^{-1}$
$A_\mathrm {OB_a}$	$2.1107 \times 10^{-1}$	$\textrm{day}^{-1}$
$D_\mathrm {OC_p}$	$2.1000 \times 10^{ 0}$	$\textrm{day}^{-1}$
$D_\mathrm {OB_p}$	$1.6570 \times 10^{-1}$	$\textrm{day}^{-1}$
$D_\mathrm {OB_u}$	$9.0000 \times 10^{-2}$	$\textrm{day}^{-1}$
$P_\mathrm {OB_p}$	$2.7138 \times 10^{-2}$	$\textrm{day}^{-1}$
$K_\mathrm {OB_p}$	$1.7543 \times 10^{-4}$	$\textrm{pM}$
$K_\mathrm {OB_u}$	$5.6328 \times 10^{-4}$	$\textrm{pM}$
$K_\mathrm {OC_a}$	$5.6328 \times 10^{-4}$	$\textrm{pM}$
$K_\mathrm {RANKL_R}^{\mathrm d}$	$5.6797 \times 10^{0}$	$\textrm{pM}$
$K_\mathrm {RANKL_R}^{\mathrm a}$	$3.4118 \times 10^{-2}$	$\textrm{pM}^{-1}$
$\textrm{RANK}$	$1.0000 \times 10^{1}$	$\textrm{pM}$
$\beta _\textrm{RANKL}$	$1.6842 \times 10^{2}$	$\mathrm {pM\ day}^{-1}$
$D_\textrm{RANKL}$	$1.0132 \times 10^{1}$	$\textrm{day}^{-1}$
$N_\mathrm {OB_p}$	$2.7030 \times 10^{6}$	$-$
$K_\textrm{PTH}^{\mathrm a}$	$1.5000 \times 10^{2}$	$\textrm{pM}$
$K_\mathrm {RANKL_O}^{\mathrm a}$	$1.0000 \times 10^{-3}$	$\textrm{pM}^{-1}$
$\beta _\textrm{PTH}$	$2.5000 \times 10^{2}$	$\mathrm {pM\ day}^{-1}$
$D_\textrm{PTH}$	$8.600 \times 10^{1}$	$\textrm{day}^{-1}$
$\mathrm {OPG^m}$	$2.0000 \times 10^{8}$	$\textrm{pM}$
$D_\textrm{OPG}$	$3.500 \times 10^{-1}$	$\textrm{day}^{-1}$
$B_\textrm{OPG}$	$1.6250 \times 10^{8}$	$\textrm{day}^{-1}$
$K_\textrm{PTH}^{\mathrm r}$	$2.2260 \times 10^{-1}$	$\textrm{pM}$
$k_{\textrm{res}}$	350	$\% \left( \textrm{pM} \, \textrm{day}\right) ^{-1}$
$\kappa$	$10^4$	$\mathrm {pM\ day}^{-1}$
$\lambda$	9.25	$-$

## Cortical bone modelling

Here, we list the pertinent (dimensionless) parameters of the one-scale approach (details in Schmidt et al. (2021)) employed for the cortical bone region of the proximal femur head, see Table 4.

**Table 4:** Pertinent Parameters of the One-Scale Approach

Parameter	Numerical Value	
$E^\textrm{s}$	10000	Solid bone Young’s modulus
$\nu ^\textrm{s}$	0.2	Solid Poisson’s ratio
$\rho ^\textrm{s}_0$	2	Solid bone density
$\bar{\rho }^\star _0$	1.8$-$0.55	Initial nominal density
$\bar{m}$	3	Exponent
$\bar{n}$	2	Exponent
$\bar{N}$	2	Homogenisation exponent
$\bar{c}$	1.2	Remodelling velocity factor
$\bar{\psi }_{0}^\textrm{a}$	0.0033	Attractor stimulus

## Footnotes

1 Note that we use bold upright font to denote matrices and vectors that merely serve to collect variables and parameters to distinguish them from tensors. In matrix–vector notation, we do not use dots for symbolising multiplication and indicate transposing a matrix or referring to a row vector by $^\textrm{T}$.

2 RANKL abbreviates the Receptor Activator of Nuclear factor $\underline{\kappa } _\beta$ Ligand.

3 The computational cost of our three-scale approach is essentially due to three intermingled factors: (1) the finite element discretisation density at the macro scale, (2) the modelling complexity of the ZVE capturing the trabecular architecture at the meso scale, and (3) the time integration of the BCPM at the cellular scale. Since (3) is solved in multiple, i.e. O($10^2$) cellular time steps for the O($10^0-10^2$) truss elements in the ZVE of (2) and for each of the O($10^1-10^2$) macro=meso time steps, (2) is incorporated into each of the O($10^3$) quadrature points of (3), and (3) is solved by an incremental-iterative solution scheme with O($10^0$) iterations in each of the O($10^1-10^2$) macro time steps, the overall cost scales multiplicatively with the spatio-temporal resolution accuracy on each scale. The current implementation of (1)-(3) has not yet been optimised for computational efficiency, thus a fair quantitative specification of the run time for the various examples is cumbersome. On a standard laptop the current Matlab implementation took O($10^0$) minutes for the examples discussed in Sect. 3.1 and O($10^3$) minutes for the larger example in Sect. 3.2. However, these run times are not objective and will be shortened in the future by a more cost-aware implementation.
